# Ion Mobility Mass Spectrometry Uncovers Guest‐Induced Distortions in a Supramolecular Organometallic Metallosquare

**DOI:** 10.1002/anie.202100914

**Published:** 2021-06-10

**Authors:** Cristian Vicent, Victor Martinez‐Agramunt, Viraj Gandhi, Carlos Larriba‐Andaluz, Dmitry G. Gusev, Eduardo Peris

**Affiliations:** ^1^ Institute of Advanced Materials (INAM) Universitat Jaume I Av. Vicente Sos Baynat s/n 12071 Castellón Spain; ^2^ Servei Central d'Instrumentació Científica (SCIC) Universitat Jaume I Avda. Sos Baynat s/n 12006 Castellón Spain; ^3^ Department of Mechanical and Energy Engineering IUPUI Indianapolis IN 46206 USA; ^4^ Department of Chemistry and Biochemistry Wilfrid Laurier University 75 University Avenue West Waterloo Ontario N2L 3C5 Canada

**Keywords:** host–guest chemistry, induced-fit distortions, ion mobility mass spectrometry (IM-MS), palladium, supramolecular organometallic complexes (SOCs)

## Abstract

The encapsulation of the tetracationic palladium metallosquare with four pyrene‐bis‐imidazolylidene ligands [**1**]^4+^ with a series of organic molecules was studied by Electrospray ionization Travelling Wave Ion‐Mobility Mass Spectrometry (ESI TWIM‐MS). The method allowed to determine the Collision Cross Sections (CCSs), which were used to assess the size changes experienced by the host upon encapsulation of the guest molecules. When fullerenes were used as guests, the host is expanded ΔCCS 13 Å^2^ and 23 Å^2^, for C_60_ or C_70_, respectively. The metallorectangle [**1**]^4+^ was also used for the encapsulation of a series of polycyclic aromatic hydrocarbons (PAHs) and naphthalenetetracarboxylic diimide (NTCDI), to form complexes of formula [(NTCDI)_2_(PAH)@**1**]^4+^. For these host:guest adducts, the ESI IM‐MS studies revealed that [**1**]^4+^ is expanded by 47–49 Å^2^.. The energy‐minimized structures of [**1**]^4+^, [C_60_@**1**]^4+^, [C_70_@**1**]^4+^, [(NTCDI)_2_(corannulene)@**1**]^4+^ in the gas phase were obtained by DFT calculations.Introduction

## Graphical abstract

We present an ion‐mobility mass spectrometry (IM‐MS) study on the encapsulation characteristics of a shape‐adaptable organometallic metallocage, and demonstrate that IM‐MS in combination with DFT calculations constitutes a reliable approach to quantify distortions experienced by the host upon guest encapsulation.

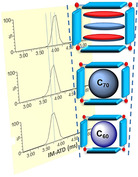

Described by Koshland in 1958,^[1]^ induced‐fit is a molecular recognition mechanism used by Nature to attain a tight binding between a molecular host (often an enzyme) and a guest, to confer allosteric regulation through conformational changes upon binding.^[2]^ Such induced‐fit conformational changes can be used for maximizing the host‐guest interactions and consequently is a fundamental strategy for constructing effective artificial receptors.^[3]^ Supramolecular coordination complexes (SCCs),^[4]^ feature well‐defined cavities prone to guest encapsulation, and different shapes and sizes can be attained given the almost unlimited combination of ligands and metals that can be used to construct metallosupramolecular assemblies. However, the lack of flexibility of the “well‐defined” shapes and sizes of the cavities of the artificial hosts make their conformational changes upon guest binding smaller than those shown in biological receptors. X‐ray diffraction and NMR techniques are the most widely used tools for studying the conformational changes experienced by a host upon guest binding.^[5]^ Obviously, the most accurate picture of the induced‐fit changes can be obtained when the structures of the free (empty) host and the host‐guest adduct can be compared, i.e., when the single crystal X‐ray diffraction studies can be performed. However, this approach fails sometimes because the solid‐state structures may differ greatly from the structures of the same species in solution or in the gas phase. In this context, Ion‐mobility mass spectrometry (IM‐MS) is gaining popularity as a new member of the structural analysis toolkit used in supramolecular chemistry.^[6]^

Ion mobility MS separates gas‐phase ions (typically generated by ESI) by allowing them to drift under the influence of an electric field against a buffer gas. The drift times measured in IM‐MS depend on the ion collision cross sections (CCS) and can be ultimately correlated to ion size and shape. Examples of the use of the IM‐MS technique have been reported for supramolecular coordination complexes (SCCs) supported by N‐, O‐ donor, Werner‐type ligands, and include topological characterization studies^[7]^ and the identification of geometric isomers.^[8]^ Furthermore, recent studies showed that IM‐MS could be used to visualize the expansion and contraction of chiral palladium cages upon addition of different guests.[^7l^, ^7m^]

Supramolecular organometallic complexes (SOCs)^[9]^ are gaining popularity among SCCs due to the availability of an increasing number of N‐heterocyclic carbene (NHC) polydentate ligands.^[10]^ During the last three years, we focused our attention on the preparation of NHC‐based SOCs that we used for the recognition of a variety of organic molecules.^[11]^ In the course of these studies, we developed a series of three‐dimensional metallocages^[12]^ and two‐dimensional metallo‐boxes,^[13]^ capable of adapting their shapes to the encapsulated guests of different size. In particular, we described a size‐flexible palladium‐cornered metallosquare, based on a pyrene‐bis‐imidazolylidene ligand ([**1**](BF_4_)_4_ in Scheme 1), whose cavity size was adapted to the size of the encapsulated fullerenes (C_60_ or C_70_).^[13c]^ This change was achieved by a guest‐induced compression or expansion of the structure, and by the bending of the pyrene moieties to maximize the face‐to‐face overlap with the convex surface of the fullerenes. The same host [**1**]^4+^ was then used for the encapsulation of series of three‐stacked heteroguests,^[13b]^ but unfortunately, we could not obtain structural information about the conformational changes experienced by the host upon the encapsulation of the guests. Inspired by these findings, herein we describe the use of Electrospray ionization Travelling wave ion‐mobility mass spectrometry (ESI TWIM‐MS), combined with CCS numerical calculations from DFT‐derived structures, for detecting the expansion/compressions produced in the metallosquare [**1**](BF_4_)_4_ upon the encapsulation of fullerenes (C_60_ and C_70_), and three‐stacked planar heteroguests. We consider that the shape adaptability, robustness, solubility in ESI‐compatible solvents and its intrinsic 4+ charge, make [**1**](BF_4_)_4_ an optimum candidate for investigating its guest‐induced distortions by ESI IM‐MS.

**Scheme 1:**
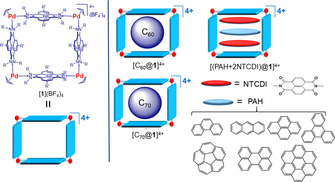
Schematic representation of metallosquare [**1**]^4+^ and its related encapsulated host:guest complexes with fullerenes (C_60_ or C_70_) and three‐stacked heteroguests.

## Results and Discussion

The ESI mass spectrum of 1 μM solutions of [**1**](BF_4_)_4_ in acetonitrile shows the base peak at *m*/*z* 766.4, assigned to [**1**]^4+^. A lower intensity peak at *m*/*z* 1050.6 due to [**1** + BF_4_]^3+^ is also observed. Lower nuclearity species were not detected neither as a result of gas‐phase fragmentation of [**1**]^4+^ upon ESI, nor via its degradation in solution when the ESI mass spectrum was recorded after several days. This observation indicates that [**1**]^4+^ is stable at the μM‐concentrations used in the ESI experiment. The ESI IM mass spectrum of [**1**]^4+^ (see SI) is identical to that found by single‐stage ESI‐MS. The experimental isotopic pattern of [**1**]^4+^ perfectly matches the simulation, thus the presence of isobaric [Pd_2_L_2_]^2+^ or [Pd_3_L_3_]^3+^ species formed by heating or fragmentation upon IM conditions can be discarded.

**Figure 1:**
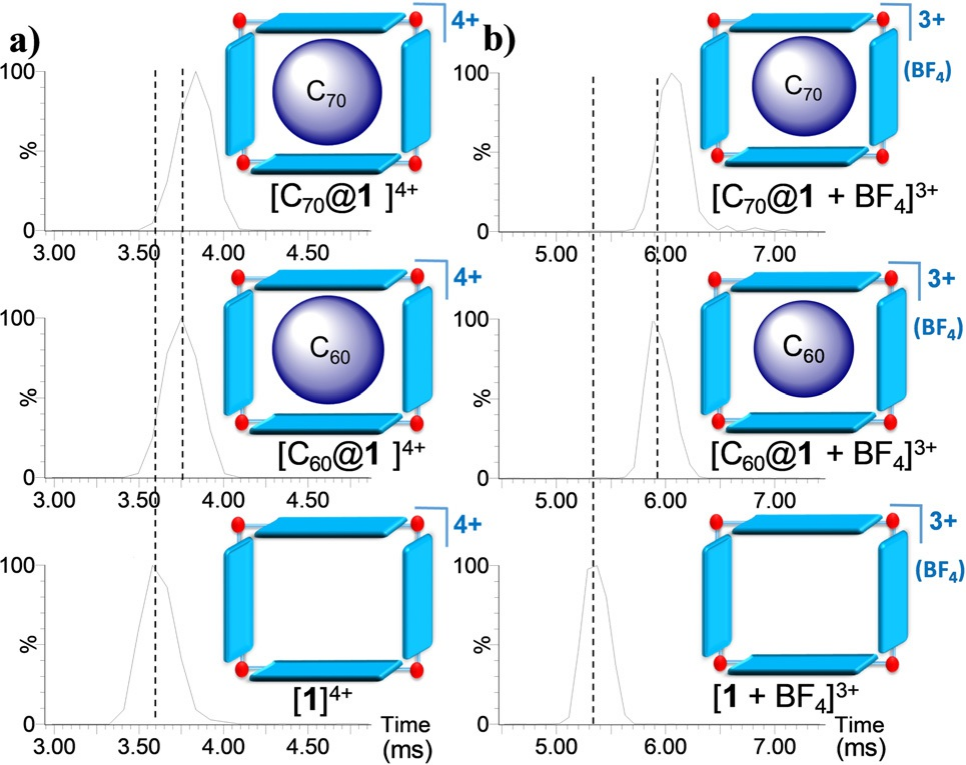
Ion mobility arrival time distributions (ATDs) for: a) [**1**]^4+^ (*m*/*z* 766.4; bottom), [C_60_@**1**]^4+^ (*m*/*z* 946.4; middle) and [C_70_@**1**]^4+^ (*m*/*z* 976.4; top) ions, and b) [**1** + BF_4_]^3+^ (*m*/*z* 1050.6; bottom), [C_60_@**1** + BF_4_]^3+^ (*m*/*z* 1290.6; middle) and [C_70_@**1** + BF_4_]^3+^ (*m*/*z* 1330.2; top) ions. Broken black vertical lines are used as a visual guide of the shift experienced by the different species.

As we showed previously, the encapsulation of fullerenes (C_60_ or C_70_) could be readily performed by sonication of [**1**](BF_4_)_4_ and the corresponding fullerene to yield [C_60_@**1**](BF_4_)_4_ and [C_70_@**1**](BF_4_)_4_.^[13c]^ The ESI and ESI IM mass spectra of the resulting solutions have a very similar pattern (see Figures S4 and S5 in the SI file for details), thus indicating that their identity is preserved under IM conditions. The Ion mobility arrival time distributions (ATDs) for [C_60_@**1**]^4+^ (*m*/*z* 946.4) and [C_70_@**1**]^4+^ (*m*/*z* 976.4) together with that of [**1**]^4+^ (*m*/*z* 766.4) are shown in Figure 1.

A common feature inferred from the ion mobility ATDs shown in Figure 1 is that all species display similar narrow, gaussian‐shaped arrival time distributions, in agreement with a low conformation dispersity. Moreover, the close drift times observed for the empty host [**1**]^4+^ and the fullerene adducts, indicate that the [fullerene@**1**]^4+^ complexes are truly capsular assemblies in the gas‐phase, and that [**1**]^4+^ and [fullerene@**1**]^4+^ share similar topology. Nevertheless, a small increase in the drift time (t_D_) is observed upon fullerene encapsulation (see Table 1 for values) compared to the drift time of the empty cage [**1**]^4+^. Drift time values can be converted into CCS values following the Ruotolo procedure.^[14]^ Table 1 collects the drift times and their corresponding CCS values for the compounds of this study.

**Table 1:** Drift times and experimental ^TW^CCS_N2_ values of the supramolecular complexes under study.

Entry	Compound	Drift time (ms)^[a]^	^TW^CCS_N2_ [Å^2^]^[b]^
1	[**1**]^4+^	3.63	707
2	[C_60_@**1**]^4+^	3.75	720
3	[C_70_@**1**]^4+^	3.84	730
4	[(NTCDI)_2_ (triphenylene)@**1**]^4+^	4.00	744
5	[(NTCDI)_2_ (pyrene)@**1**]^4+^	4.00	744
6	[(NTCDI)_2_ (coronene)@**1**]^4+^	4.01	745
7	[(NTCDI)_2_ (phenanthrene)@**1**]^4+^	4.01	745
8	[(NTCDI)_2_ (anthracene)@**1**]^4+^	4.01	745
9	[(NTCDI)_2_ (perylene)@**1**]^4+^	4.00	744
10	[(NTCDI)_2_ (corannulene)@**1**]^4+^	4.02	746

[a] Samples were measured by triplicate, and standard deviations were below 0.5 %. [b] Values obtained by calibrating the drift time scale of the TWIM device with standards of known ^DT^CCS_N2_ cross‐sectional data from the literature.^[15] TW^CCS_N2_ refers to the determined CCS values using a TWIM‐MS instrument and nitrogen as buffer gas.

As can be observed from the data shown in Table 1, the CCS values increase in the order [**1**]^4+^ (707 Å^2^) < [C_60_@**1**]^4+^ (720 Å^2^) < [C_70_@**1**]^4+^ (730 Å^2^).^[16]^ As will be explained below, this trend can be rationalized by exploring the gas‐phase DFT optimized geometries (vide infra). An inspection of the triply‐charged series formed by adduction with BF_4_^−^ revealed a similar trend as that found for the quadruply‐charged analogues (see Figure 1 b) and Table S3 in the SI). The ^TW^CCS_N2_ enlargement of [C_70_@**1** + BF_4_]^3+^ with respect to [C_60_@**1** + BF_4_]^3+^ is close to 10 Å^2^.

We showed recently that metallosquare [**1**]^4+^ is effective for the simultaneous encapsulation of three large π‐conjugated heteroguests, enabling the formation of quintuple D‐A‐D‐A‐D (D=donor, A=acceptor) stacks (see Scheme 1).^[13b]^ The supramolecular quaternary [(NTCDI)_2_(PAH)@**1**]^4+^ complexes (NTCDI=naphthalenetetracarboxylic diimide; PAH=polycyclic aromatic hydrocarbon=pyrene, triphenylene and coronene) were formed by the direct mixing of the components. Herein, we also performed experiments using phenanthrene, anthracene, perylene and corannulene, to widen the scope of the study. Complexation‐induced ^1^H chemical shift changes were observed in the NMR spectra, consistent with the encapsulation occurring in solution (see Figures S21–S24). In all cases, the ESI mass spectra displayed prominent supramolecular peaks assigned to [(NTCDI)_2_(PAH)@**1**]^4+^. However, switching to the IM‐MS mode resulted in a dramatic reduction of the peak abundances of these supramolecular aggregates, which became barely detectable, in most cases. This observation is strongly suggestive of a labile nature of the [(NTCDI)_2_(PAH)@**1**]^4+^ complexes, compared to those of the fullerenes, for which both single‐stage and ESI IM mass spectra were identical. Similar conclusions can be drawn from gas‐phase fragmentation studies of the isolated [(NTCDI)_2_(PAH)@**1**]^4+^ and [(fullerene)@**1**]^4+^ ions by collision‐induced dissociation (CID) experiments. The fullerene‐encapsulated ions [(fullerene)@**1**]^4+^ remained largely unchanged under CID conditions. Conversely, the series of [(NTCDI)_2_(PAH)@**1**]^4+^ cages dissociated similarly and produced characteristic fragment ions due to eliminations of the guests under identical conditions (see Figures S12–S20). Energy resolved CID experiments (see breakdown graphs in SI) indicate that guest eliminations occur simultaneously.

**Figure 2:**
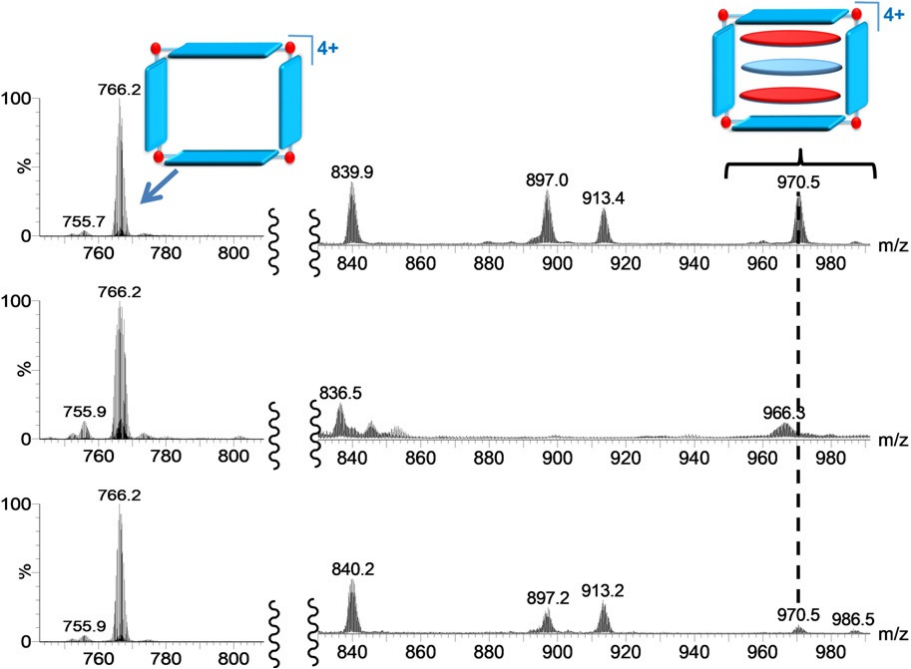
Single‐stage ESI mass spectrum (top) and ESI‐IM mass spectra using 45 (middle) and 35 V (bottom) trap bias potentials, respectively. The 830 to 930 *m*/*z* range has been enhanced 10‐fold. The peak at *m*/*z* 970.5 corresponds to [(NTCDI)_2_(triphenylene)@**1**]^4+^.

Fragmentation or isomerization of fragile molecules due to heating within the TWIM‐MS drift tube is a well‐documented phenomenon.^[17]^ Labile metallocages are also prone to dissociation under IM‐MS conditions.^[7g]^ In our studies, the use of gentle ESI source conditions, together with tuning the ion transmission in the TWIM‐MS mode were of major importance for keeping the [(NTCDI)_2_(PAH)@**1**]^4+^ aggregates below their dissociation threshold. Figure 2 illustrates the importance of ion transmission adjustment for the detection and characterization of [(NTCDI)_2_(PAH)@**1**]^4+^ assemblies, exemplified for the case of [(NTCDI)_2_(triphenylene)@**1**]^4+^.

**Figure 3:**
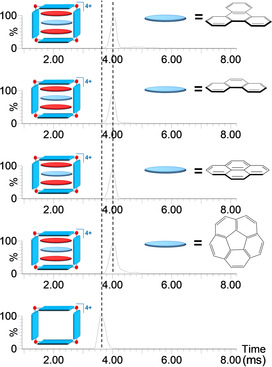
Ion mobility arrival time distributions for selected ions [**1**]^4+^ (*m*/*z* 766.4; bottom) and the series of quaternary ions [(NTCDI)_2_(PAH)@**1**]^4+^ (PAH=triphenylene, anthracene, pyrene and corannulene).

Under the optimized conditions, the whole series of quaternary [(NTCDI)_2_(PAH)@**1**]^4+^ cationic assemblies was studied by ESI IM‐MS. Again, a common feature inferred from the ion mobility ATDs shown in Figure 3, is that the narrow Gaussian‐shaped arrival time distributions are strongly suggestive of low conformation dispersity. As can be seen from the data shown in Table 1, they all display identical ATD values, regardless of the nature of the π‐donor encapsulated guest. The estimated CCS values are in the narrow 744–746 Å^2^ range. These similarities indicate that the π‐donor PAH guests, while sandwiched between the two π‐acceptor NTCDI molecules, are embedded within the cavity of [**1**]^4+^ and show overall similar dimensions of the supramolecular assembly.

The complexation of corannulene is singular because its bowl‐shaped nature renders a less effective interaction with planar polyaromatic donors, such as NTCDI.^[18]^ This explains why this curved PAH normally displays lower binding affinities compared to its planar 20‐electron analogue, perylene.[^13a^, ^19^] When corannulene was mixed with NTCDI in the presence of [**1**]^4+^, the ^1^H NMR spectrum displayed broad signals, which indicated labile encapsulation (see the ^1^H NMR spectrum in Figure S20 in the SI). However, ESI‐IM‐MS provided convincing evidence that the host‐guest [(NTCDI)_2_(corannulene)@**1**]^4+^ was formed, and its composition and topology could be assessed by comparing with the series of planar π‐donors. Although the incorporation of this curved PAH could be expected to expand the volume of the metallosquare host, the observed CCS value of 746 Å^2^ was practically identical to the values obtained for the other [(NTCDI)_2_(PAH)@**1**]^4+^ species in Figure 3. An intuitive interpretation of this result is that the molecule of corannulene may be flattened upon encapsulation, but the detailed reasons that explain this observation were elucidated from the DFT analysis of the structures (see below).

**Figure 4:**
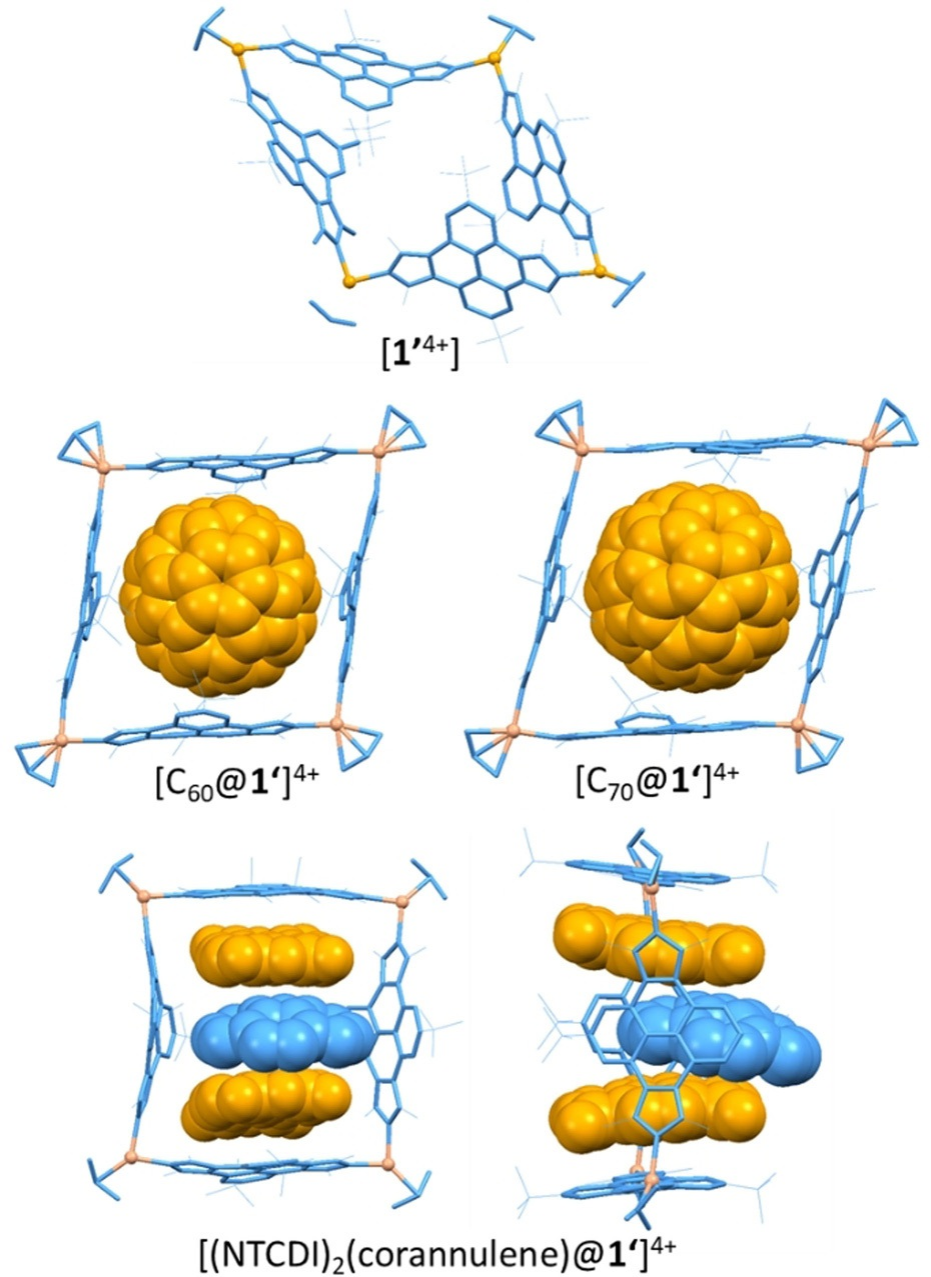
DFT optimized structures of [**1′**]^4+^, [C_60_@**1′**]^4+^ [C_70_@**1′**]^4+^ and [(NTCDI)_2_(corannulene)@**1′**]^4+^ (in two perspectives).

In order to attain an accurate picture of the distortions suffered by [**1**]^4+^ with the whole series of encapsulated guests, we performed DFT (MN15‐L/Def2SVP) calculations of [**1**]^4+^, [C_60_@**1**]^4+^ and [C_70_@**1**]^4+^ in gas phase. Due to its intriguing nature, we also calculated the structure of [(NTCDI)_2_(corannulene)@**1**]^4+^. To simplify the calculations and to avoid considering a large number of conformational isomers, we substituted the *n*Bu groups bound to the nitrogen atoms of the imidazolylidenes by methyl groups. Figure 4 shows the resulting calculated structures where [**1**′]^4+^ denotes the modified host, possessing all NMe groups.

The structure of [**1′**]^4+^ in the gas phase displays a distorted arrangement from an ideal square. Shrinkage from opposite corners of the square leads to a rhombohedral‐shaped molecule (see Figure 4, top), in which the pyrene moieties are significantly bent towards the interior of the cavity. This situation clearly contrasts with the X‐ray solid state structure reported for [**1**]^4+^(BF_4_^−^)_4_, which showed an almost perfect square‐shaped geometry for [**1**]^4+^. The difference can be ascribed mainly to the presence of three molecules of solvent (DMF) and three counter‐anions (BF_4_^−^) in the interior of the cavity of the supramolecular host in the solid‐state structure, which are obviously absent in the gas‐phase cation. The encapsulation of C_60_ and C_70_ does not leave room within the cavity for compression and consequently, the gas‐phase structures are rather similar to those found in the solid‐state, although both show a rhombohedral shape. For the case of [C_70_@**1′**]^4+^, the C_70_ molecule displays its longer axis parallel to the *C*_2_ axis of the molecule, therefore minimizing the steric congestion in the cage. The structure of [(NTCDI)_2_(corannulene)@**1′**]^4+^ shows that the corannulene guest is sandwiched between the two NTCDI molecules. The latter are significantly bent, most likely for maximizing the π‐stacking interaction with the bowl‐shaped guest. Interestingly, the centroid of the corannulene molecule is off the axis defined by the centroids of the opposite pyrene moieties of the cage, which also contain the centroids of the NTCDI guests (see Figure 4). Related to this situation, the pyrene panels that are perpendicular to the three guests are tilted in a manner that leaves a wider space on one of the portals of the molecule, so that the steric congestion around the corannulene guest is minimized. Finally, it is important to mention that the bowl‐depth of the encapsulated corannulene molecule is 0.78 Å, therefore flattened by 0.12 Å compared to the corannulene structure in the gas phase (0.90 Å).

**Figure 5:**
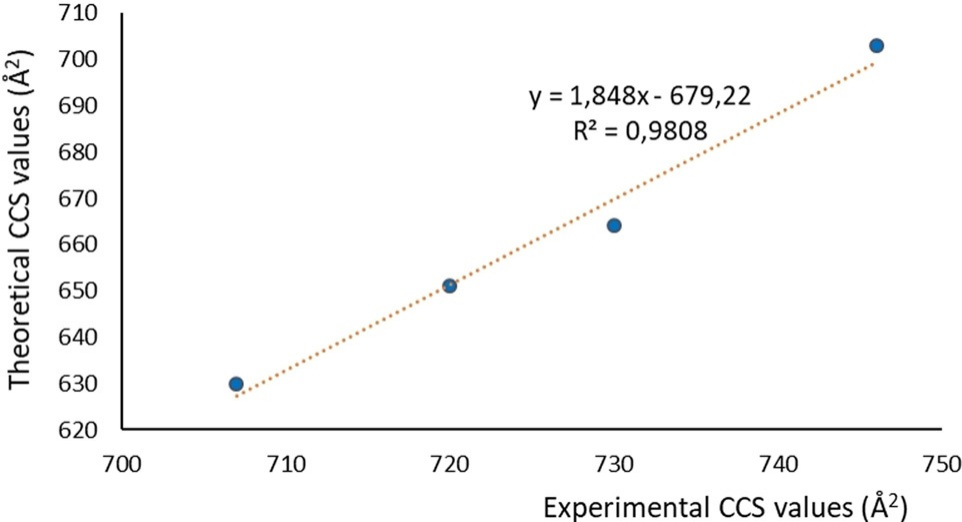
Linear regression plot showing the correlation between the experimental CCS and theoretical CCS values calculated by TM method for [**1′**]^4+^, [C_60_@**1′**]^4+^, [C_70_@**1′**]^4+^ and [(NTCDI)_2_(corannulene)@**1′**]^4+^.

The DFT‐optimized structures were used as inputs for CCS predictions, and then these were compared with the experimental data obtained by IM‐MS. The CCS values for [**1′**]^4+^, [C_60_@**1′**]^4+^, [C_70_@**1′**]^4+^ and [(NTCDI)_2_(corannulene)@**1′**]^4+^ were calculated using Trajectory Methods (TM), implemented in the IMoS software.^[20]^ The calculated structures of the model [**1′**]^4+^ cation and its respective host:guest adducts display predicted CCS values (630 Å^2^ for [**1′**]^4+^, 651 Å^2^ for [C_60_@**1′**]^4+^, 664 Å^2^ for [C_70_@**1′**]^4+^ and 703 Å^2^ for [(NTCDI)_2_(corannulene)@**1′**]^4+^) that are consistently smaller than the experimental ones. This is a consequence of the use of the small NMe instead of NBu groups in the calculated structures. The structural differences are linearly correlated as illustrated in Figure 5 where the experimental CCS values are plotted against the calculated data.

## Conclusion

Our results demonstrate that IM‐MS in combination with DFT modeling and CCS predictions is a reliable tool for assessing guest‐induced host distortions on the basis of their CCS values. The CCS data also serve to illuminate the exceptional size adaptability of [**1**]^4+^, which is able to expand by ΔCCS 37–39 Å^2^ from its empty form to accommodate the three guests in [(NTCDI)_2_(PAH)@**1**]^4+^. Equally interesting is the evidence that ESI IM‐MS may also be useful for detecting distortions experienced by guests upon encapsulation, as we showed for the case of the flattening of corannulene. This result is remarkable, especially if we take into account that the flattening of corannulene upon encapsulation inside a molecular host has been observed experimentally very few times,[^13a^, ^19^, ^21^] but in all these cases such distortions were only evidenced by means of X‐ray diffraction studies. Since IM‐MS can be registered from small amounts, the only pre‐requisite is handling ESI‐amenable compounds. In this regard, it is also important to note that [**1**]^4+^ constitutes an excellent prototype for this type of studies, due to the high stability conferred by the di‐NHC linkers, the large binding affinities with three‐ and two‐dimensional hosts, and the intrinsic 4+ charge of all the resulting host‐guest complexes. We expect that this study will inspire researchers in the field of supramolecular organometallic chemistry to incorporate the use of the IM‐MS technique to facilitate the study of structural properties such as breathable motions or guest‐induced distortions of the metallosupramolecular assemblies.

## Conflict of interest

The authors declare no conflict of interest.

## Supporting information

As a service to our authors and readers, this journal provides supporting information supplied by the authors. Such materials are peer reviewed and may be re‐organized for online delivery, but are not copy‐edited or typeset. Technical support issues arising from supporting information (other than missing files) should be addressed to the authors.

Supplementary
