# Supplementary material for: Ion Mobility Mass Spectrometry Uncovers Guest‐Induced Distortions in a Supramolecular Organometallic Metallosquare
Source: Angew Chem Int Ed Engl. 2021 Jun 10;60(28):15412–7. doi: 10.1002/anie.202100914 (PMC8361979; doi:10.1002/anie.202100914)
Supplement: Supplementary file 1 — Supplementary [file ANIE-60-15412-s001.pdf]

Angewandte Chemie
Eine Zeitschrift der Gesellschaft Deutscher Chemiker

Supporting Information

Ion Mobility Mass Spectrometry Uncovers Guest-Induced Distortions in a Supramolecular Organometallic Metallosquare

Cristian Vicent, Victor Martinez-Agramunt, Viraj Gandhi, Carlos Larriba-Andaluz, Dmitry G. Gusev, and Eduardo Peris**

anie_202100914_sm_miscellaneous_information.pdf

Table of contents:

1 Experimental Section

2 CID and ESI TWIM mass spectra and NMR of the investigated compounds

3 References

4 Cartesian coordinates for the calculated structures

1 Experimental Section

1.1 General Considerations

Compounds [**1**]$(BF_4)_4$ and its fullerene adducts, namely [C_{60}@**1**]$(BF_4)_4$ and [C_{70}@**1**]$(BF_4)_4$ were synthesized according to literature methods.[1] The formation of the quaternary $[(NTCI)_2$(PAH)@**1**]$(BF_4)_4$ (PAH = triphenylene, coronene and pyrene) compounds was carried out as reported.[2] Bovine Hemoglobine tryptic digest was purchased from waters (MassPREP Bovine Hemoglobin Standard). Angiotensin I and Melittin were purchased from Sigma Aldrich. NMR spectra were recorded on a Varian Innova 500 MHz spectrometer. All values of the chemical shift are in ppm regarding the δ-scale and referenced to the non-deuterated residual solvent.

1.2 Electrospray Ionization and Ion Mobility Mass Spectrometry

ESI and ESI IM mass spectra were performed using a SYNAPT XS High Definition Mass Spectrometer (Waters Corporation, Manchester, UK) equipped with an electrospray ionization (ESI) source. The ions generated are transmitted through the StepWave XS ion guide to the first quadrupole (Q), then to the traveling wave ion mobility (TWIM) cell, and finally analyzed with a time-of flight (TOF) mass analyzer. The ion mobility separation occurs through the so-called *triwave* device that operates with three regions: trap, ion mobility separation, and transfer with a helium cell located between the trap and ion mobility separation regions. Further details and a schematic view are given in Scheme S1.

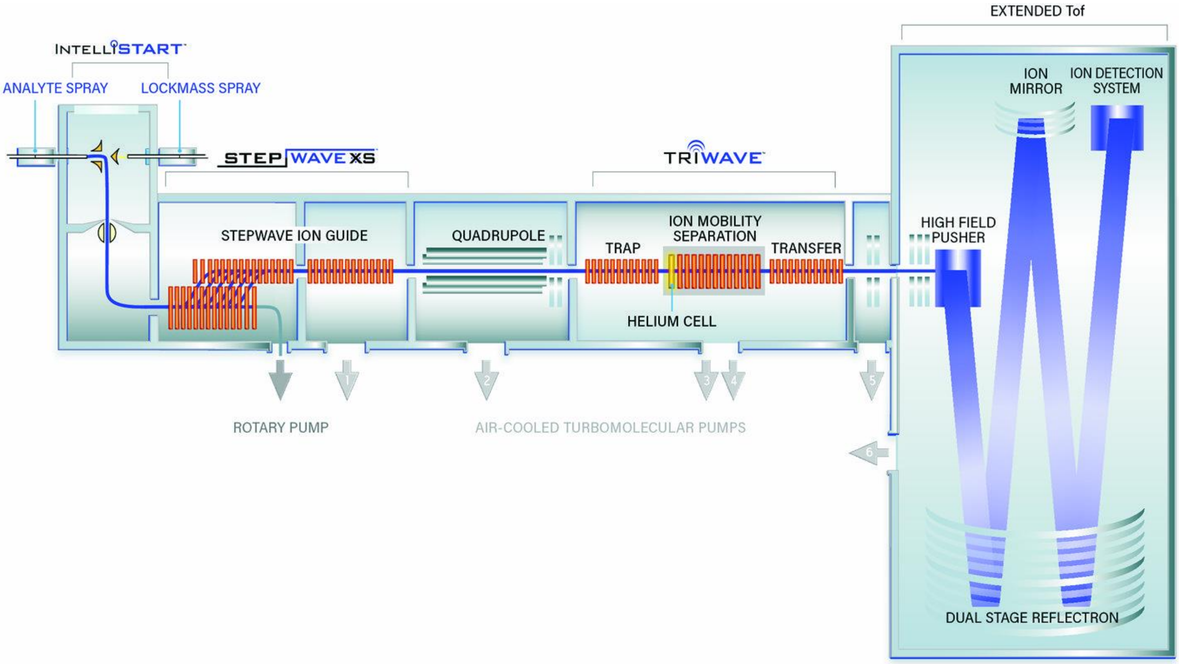

Scheme S1. Schematic view of the Synapt XS High Definition Mass Spectrometer. Reprinted from the waters.com webpage with permission from the Waters Corporation.

1.3 Single-stage ESI-MS and CID experiments. A capillary voltage was set to 1.5 kV operated in the positive ionization mode and in the resolution mode. Source settings were adjusted to keep intact the supramolecular adducts of interest. Typical values were cone voltage 20 to 40 V and source offset 4 V; source and desolvation temperatures were set to 110 and 350 ºC, respectively. Cone and desolvation gas flows were 150 and 500 (L/h), respectively. Sample solutions were prepared from stock acetonitrile 1mM solutions by 1000-fold dilution with acetonitrile to reach the 1μM concentration and introduced directly to the ESI chamber through an external syringe pump at a flow rate of 5 μL·min^{-1}. Samples were investigated over several days in the 50 to 2500 *m/z* range and fragmentation of the robust [**1**]$^{4+}$ skeleton was not observed at all. Calibration of the *m/z* axis up to *m/z* 3000 was performed using the routine implemented in intellistart from a mixture of sodium and cesium iodide (2 mgmL^{-1} in 1:1 *v/v* H_2O:isopropanol). Comparison of experimental vs theoretical isotopic pattern was carried out

using Masslynx 4.2 (SCN 982). CID experiments were performed by mass selecting the supramolecular $[(NTCDI)_2(PAH)@\mathbf{1}]^{4+}$ and the fullerene $[(fullerene)@\mathbf{1}]^{4+}$ ions of interest in the first quadrupole and increasing the collision voltage (V) in the trap region starting from 2 V and stepped by 3 V up to a maximum of 20 V. An isolation width of approximately 2 Da was selected (LM resolution set to 8). Collision energies were converted to the center-of-mass frame, E_{CM} =q*$m/(m+M)E$lab, where q stands for the charge state and *m* and *M* stand for the masses of the collision gas and the ionic species, respectively. For the breakdown profiles representations, signal intensities were obtained from the average of 20 scans and measuring the area of the fragmentation peaks. These graphs were represented taking into account the relative abundance of the precursor and product peaks of each compound (*I*precursor ion or *I*product ion / *I*precursor ion + *I* product ion) against *E*CM.

1.4 ESI TWIM-MS. The same sample solutions and source settings to that described above for single-stage ESI-MS were used. The instrument was switched from TOF acquisition to mobility TOF acquisition mode and left for 30 minutes before recording IM mass spectra. The *m/z* 50-1500 range was investigated and ion mobility separation settings were used as follows: the traveling wave height was set to 40 V and wave velocity was set to 650 m/s. The drift gas was nitrogen (N_2) at a flow rate set to 90 mL/min. The helium cell gas flow was 180.00 mL/min. IMS DC values were as follow: Entrance 20; Helium cell DC 50; Helium exit -20; Bias 3; Exit 0. Trap DC values were controlled manually; entrance, 3; trap DC bias was varied from 25 to 45 V, the 35 V value being the compromise value to visualize the intact supramolecular adducts and maximize ion transmission; Exit 0. The adjustment of the trap bias potentials (the accelerating voltage between the trap and the He cell that precedes the IMS chamber), proved to be crucial to visualize the $[(NTCDI)_2(PAH)@\mathbf{1}]^{4+}$ adducts. For example, progressive reduction of the trap bias potentials from initial 45 V to 25 V (that is, softening the ion injection conditions) allowed the $[(NTCDI)_2(donor)@\mathbf{1}]^{4+}$ species of interest to be

visualized and characterized at expenses of a significant reduction of ion abundances. All the investigated compounds and calibrants in the present work were recorded under these conditions.

The IM-MS data were processed using Masslynx 4.2 (SCN 982). All ions of interest displayed a gaussian-shaped arrival time distribution profile. Ion mobility spectra of the species of interest were extracted using a 0.15 Da mass window and were converted from waters.raw to .txt files. Gaussian fitting of the IM data was applied to improve the precision of the drift time measurements. The reported drift times values were obtained by Gaussian peak fitting using origin 6.0 (Microcal) rendering good correlation in all cases. Each sample was recorded by triplicate on the same day and the deviation in the drift time values was less than 0.5 %.

1.5 CCS Calibration: The CCS calibration protocol reported by Ruotolo was followed to convert drift times into CCS,[3] using a series of similarly charged peptide ions (Melittin, Angiotensin I and a tryptic digestion of bovine hemoglobin), which cover the transit time range of the ions of interest. Calibration of the IM-MS device for determining collision cross-sectional areas from drift time measurements was performed considering a series of multiply charged $(M + nH)^{n+}$ (n = 3 and 4) species and their $^{DT}CCS_{N2}$ values were taken from the literature (Angiotensin I, melittin[4] and tryptic digest of bovine Hemoglobine[5]). As the TWIMS device is operated with N_2 buffer gas, the obtained ^{TW}CCS values will be noted $^{TW}CCS_{N2}$. Drift times (t_D) were subjected to correction for mass-dependent and mass-independent flight times according to $t_D{}' = t_D - C * \frac{\sqrt{m/z}}{1000} - 0{,}9$ (C = 1.5 and the term 0.9 ms is the mass-independent time to account for the time of transit of one wave in the IMS and the transfer region). The literature CCS values were converted to CCS´ according to $CCS' = \frac{CCS\sqrt{\mu}}{z}$ where μ and z stands for the reduced mass of the collision partners and the charge state, respectively. The calibration curve is represented as CCS´ as a function of $t_D{}'$ using a power law,[10] $CCS' = A \times (t_D{}')^B$. Constants A and B were subsequently derived from the calibration

plot and used to calculate cross-sectional areas ($^{TW}CCS_{N2}$) of unknown species from corrected drift time measurements extracted for specific *m/z* values from the data. Table S1 and S2 collect the list of quadruply- and triply-charged IM calibrants, respectively, including sequence, charge state, *m/z*, experimental drift time, corrected drift time and published collision cross sections $^{DT}\Omega_{N2}$ (Å^2)

Table S1

**Sequence**	**Charge**	***m/z***	**t_D (ms)**	**t_D' (ms)**	**Published $^{DT}\Omega_{N2}$(Å^2)**
TYPFHFDLSHGSAQVK	4	459.3	3.15	2.22	**614**
AVEHLDDLPGALSELSDLHAHK	4	592.5	3.45	2.51	**715**
LLSHSLLVTLASHLPSDFTPAVHASLDR	4	743.3	4.52	3.58	**801**
[angiotensin I + 4H]4+	4	324.9	2.42	1.49	**549**
[melittin +4H]4+	4	712.2	4.01	3.07	**757**

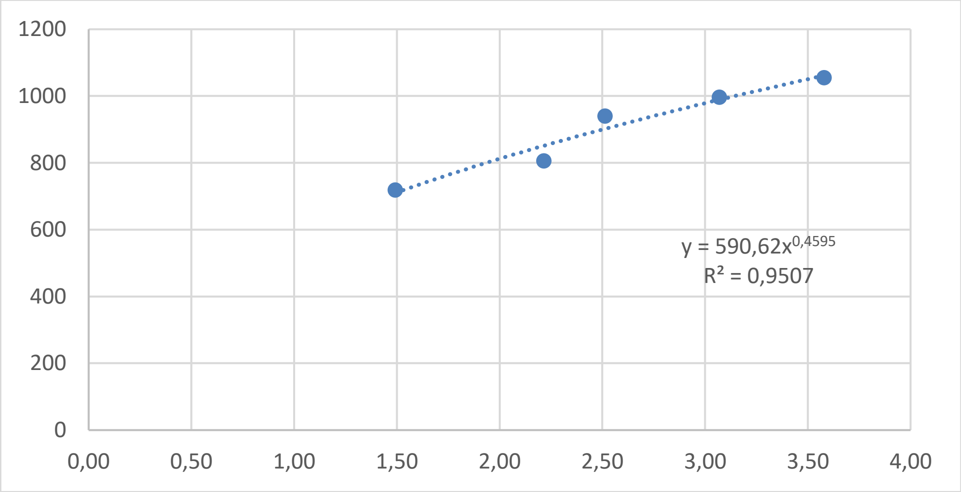

Figure S1. Plot of corrected cross sections CCS´ versus effective drift times (t_D´) for quadruply-charged calibrants. A line of best fit plotted through these points is used to obtain collision cross sections from the drift times measured for the supramolecular complexes investigated in this work.

Table S2

**Sequence**	**Charge**	***m/z***	**t_D (ms)**	**t_D' (ms)**	**Published $^{DT}\Omega_{N2}$(Å^2)**
VKVDEVGGEALGR	3	443.3	2.74	1.81	**443**
FFESFGDLSTADAVMNNPK	3	697.3	4.24	3.30	**583**
[angiotensin I + 3H]3+	3	432.9	2.96	2.03	**475**
[melittin + 3H]3+	3	949.9	6.06	5.11	**721**

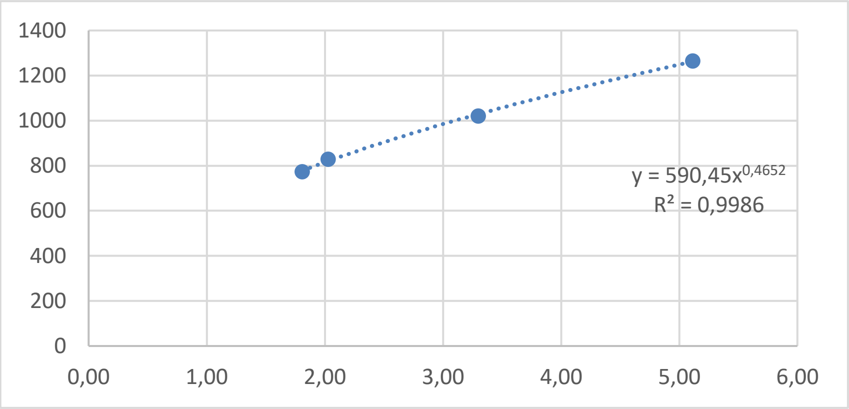

Figure S2. Plot of corrected cross sections CCS´ versus effective drift times (t_D´) for triply-charged calibrants. A line of best fit plotted through these points is used to obtain collision cross sections from the drift times measured for the supramolecular complexes investigated in this work.

Table S3. Drift times measured for the triply- and quadruply charged species and cross-sectional area extracted by comparison with a series of calibrants.

Host:guest complex	Charge	m/z	t_D (ms)	t_D' (ms)	Experimental $^{TW}CCS_{N2}$ (Å^2)
[**1**]$^{4+}$	4	766.4	3.63	2.69	707
[**1** + BF_4]$^{3+}$	3	1050.2	5.34	4.39	667
[C_{60}@**1**]$^{4+}$	4	945.9	3.75	2.80	720
[C_{60}@**1** + BF_4]$^{3+}$	3	1290.6	5.93	4.98	708
[C_{70}@**1**]$^{4+}$	4	976.9	3.84	2.89	730
[C_{70}@**1** + BF_4]$^{3+}$	3	1331.2	6.06	5.11	716
[$(NTCDI)_2$(triphenylene)@**1**]$^{4+}$	4	970.5	4.00	3.05	744
[$(NTCDI)_2$(pyrene)@**1**]$^{4+}$	4	964.0	4.00	3.05	744
[$(NTCDI)_2$(coronene)@**1**]$^{4+}$	4	988.5	4.01	3.05	744
[$(NTCDI)_2$(phenanthrene)@**1**]$^{4+}$	4	958.0	4.01	3.04	745
[$(NTCDI)_2$(anthracene)@**1**]$^{4+}$	4	958.0	4.01	3.04	745
[$(NTCDI)_2$(perylene)@**1**]$^{4+}$	4	976.5	4.00	3.04	744
[$(NTCDI)_2$(corannulene)@**1**]$^{4+}$	4	976.0	4.02	3.07	746

1.6 NMR studies on host-guest adduct formation with PAHs.

The formation of the quaternary supramolecular [$(NTCI)_2$(PAH)@**1**]$(BF_4)_4$ (PAH = anthracene, phenanthrene, perylene and corannulene) was performed by adapting the previous synthetic protocol described for other PAHs.[2] Typically, to an NMR tube containing a 1mM CD_3CN solution of host [**1**]$(BF_4)_4$, two equivalents of the Polycyclic Aromatic Hydrocarbon and then two equivalents of NTCDI were added. The resulting suspensions were placed for 30 minutes

in the ultrasonic bath before recording the spectra. Representative NMR spectra are given in the Supplementary Information file.

1.7 Trajectory method (TM) CCS predictions.

To compare empirical CCS results to DFT-derived structures, IMoS was used to calculate the average drag caused by the impinging gas molecules over the flight path. The potentials employed in this case correspond to those of the standard TM methods using a 4-6-12 potential. How these calculations are performed is available elsewhere.[6]

1.8 Computational Details.

All calculated complexes of this paper are diamagnetic and possess a +4 net charge. The gas-phase geometry optimizations were carried out with Gaussian 16, rev. c.01,[7] using the MN15-L functional[8] and the def2-SVP basis set (with def2 ECP for Pd) together with the W06 density fitting basis set.[9]

2 ESI IM mass spectra, CID mass spectra and NMR of the investigated compounds

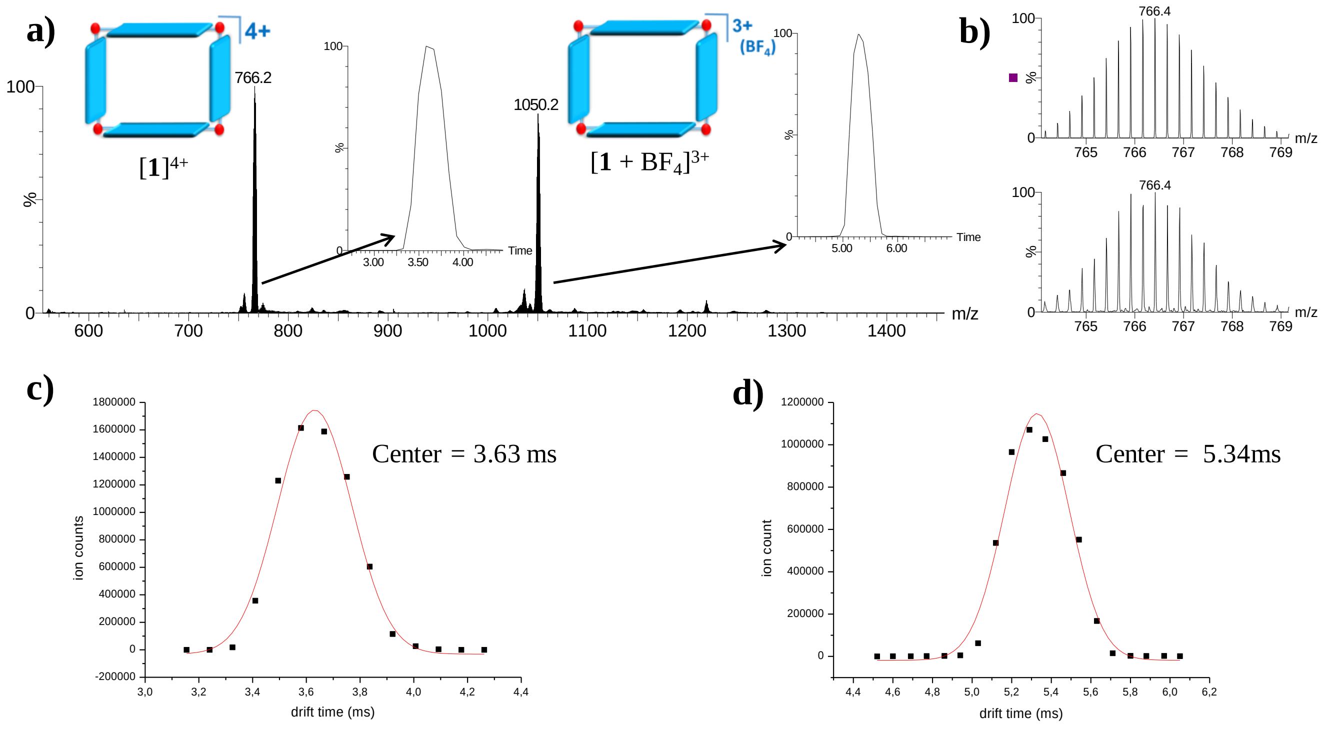

Figure S3. a) ESI TWIM mass spectrum of acetonitrile 1μM solution of compound [**1**](BF_4)$_4$ recorded in the 500 to 1500 range. The insets show the arrival time distribution for the most abundant isotopomer (766.4 and 1050.2 for [**1**]$^{4+}$ and [**1** + BF_4]$^{3+}$, respectively) using an absolute window of 0.15 Da; b) comparison between the experimental (bottom region) and theoretical (upper region) isotopic pattern of [**1**]$^{4+}$ (molecular composition $C_{180}H_{252}N_{16}Pd_4$). Gaussian fit of each arrival time distribution for the [**1**]$^{4+}$ species c) and [**1** + BF_4]$^{4+}$ d).

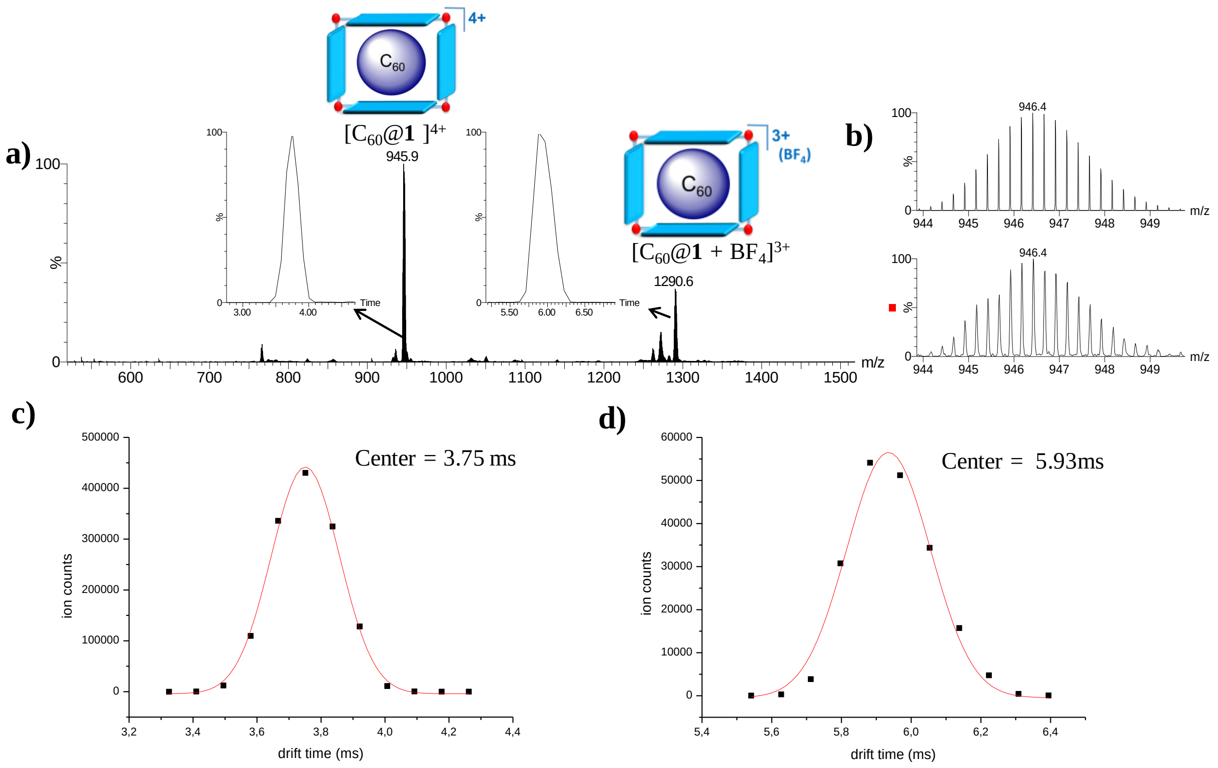

Figure S4. a) ESI TWIM mass spectrum of acetonitrile 1µM solution of compound $[C_{60}@\mathbf{1}](BF_4)_4$ recorded in the 500 to 1500 range. The insets show the arrival time distribution for the most abundant isotopomer (945.9 and 1290.6 for $[C_{60}@\mathbf{1}]^{4+}$ and $[C_{60}@\mathbf{1} + BF_4]^{3+}$, respectively) using an absolute window of 0.15 Da; b) comparison between the experimental (bottom region) and theoretical (upper region) isotopic pattern of $[C_{60}@\mathbf{1}]^{4+}$ (molecular composition $C_{240}H_{252}N_{16}Pd_4$). Gaussian fit of each arrival time distribution for the $[C_{60}@\mathbf{1}]^{4+}$ species c) and $[C_{60}@\mathbf{1} + BF_4]^{4+}$ d).

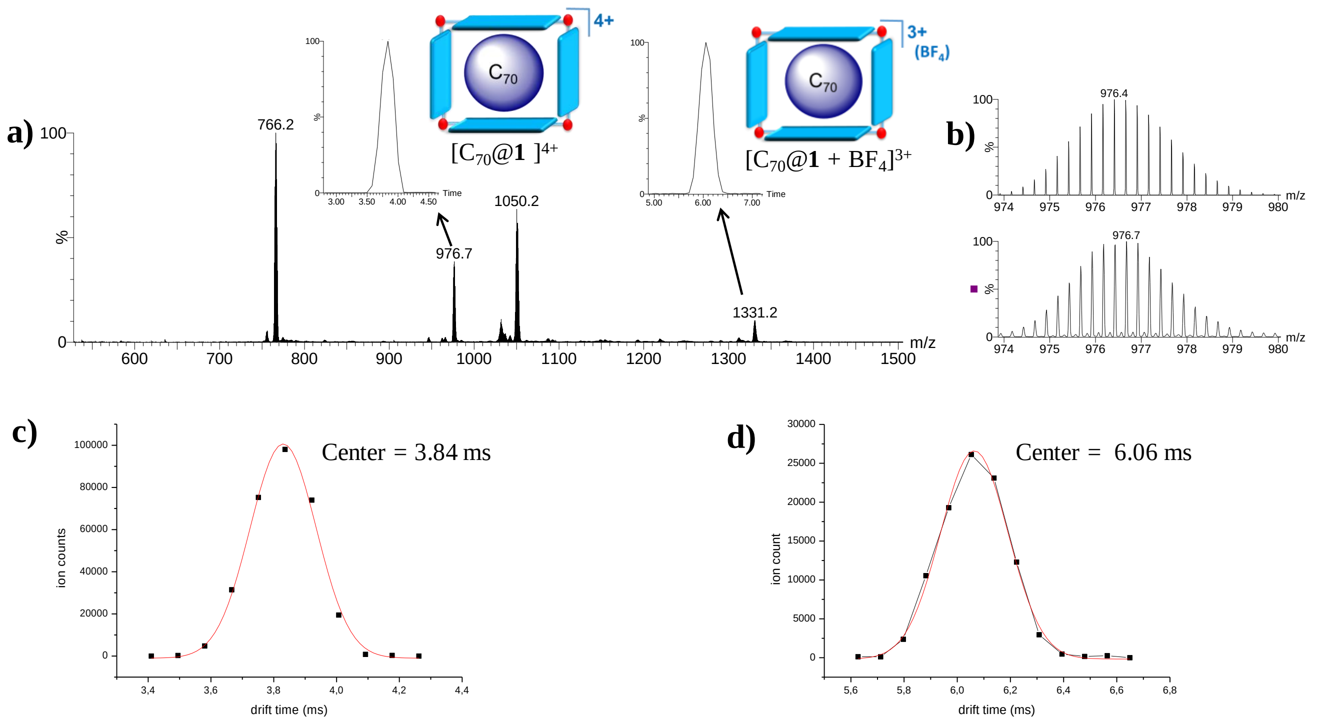

Figure S5. a) ESI TWIM mass spectrum of acetonitrile 1μM solution of compound $[C_{70}@\mathbf{1}](BF_4)_4$ recorded in the 500 to 1500 range. The insets show the arrival time distribution for the most abundant isotopomer (976.9 and 1331.2 for $[C_{70}@\mathbf{1}]^{4+}$ and $[C_{70}@\mathbf{1} + BF_4]^{3+}$, respectively) using an absolute window of 0.15 Da. b) comparison between the experimental (bottom region) and theoretical (upper region) isotopic pattern of $[C_{70}@\mathbf{1}]^{4+}$ (molecular composition $C_{250}H_{252}N_{16}Pd_4$). Gaussian fit of each arrival time distribution for the $[C_{70}@\mathbf{1}]^{4+}$ species c) and $[C_{70}@\mathbf{1} + BF_4]^{4+}$ d).

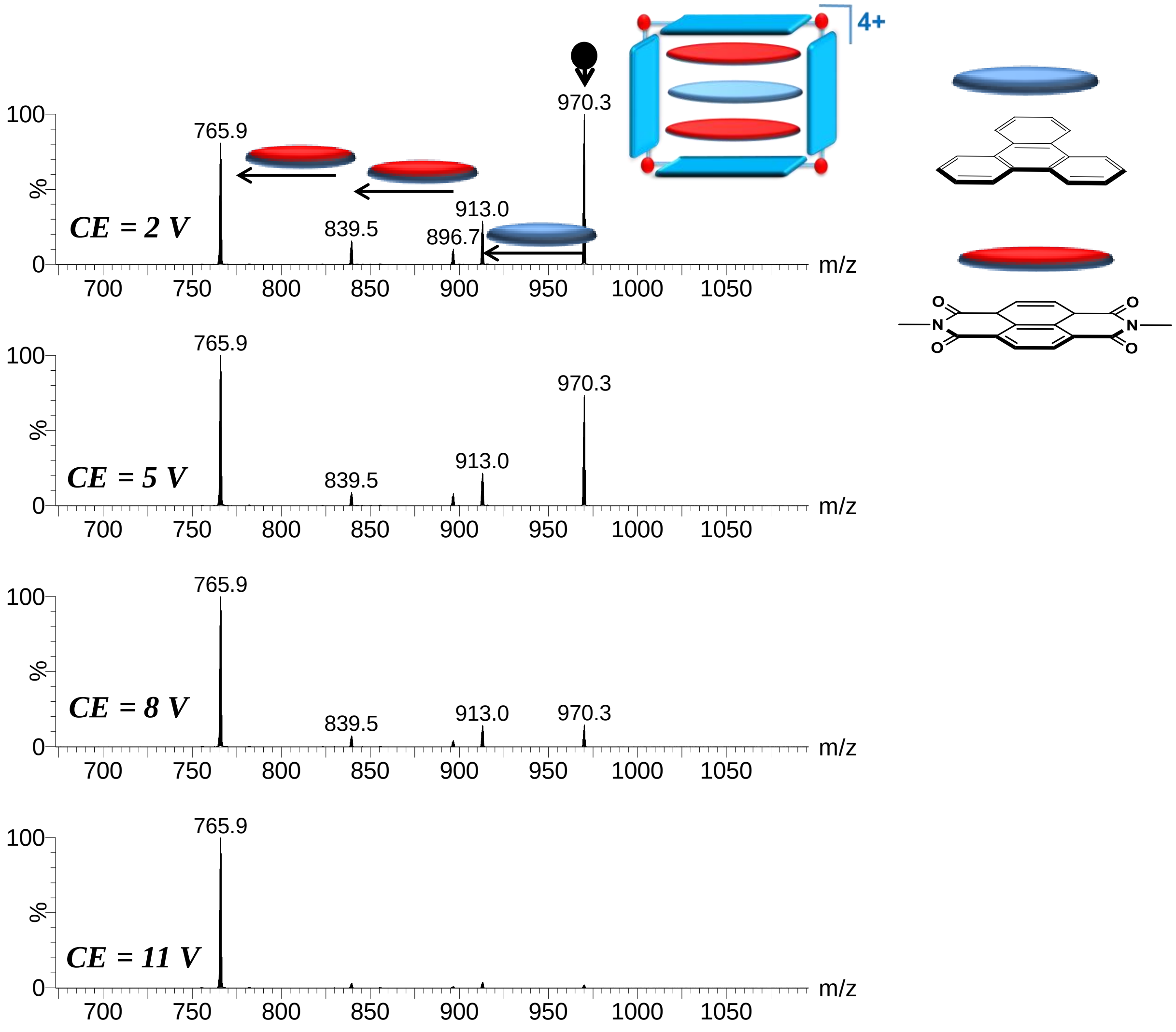

Figure S6. CID mass spectra of the supramolecular [(NTCDI)$_2$(triphenylene)@**1**]$^{4+}$ recorded at increasing collision energies from 2 to 11 V from the top to the bottom.

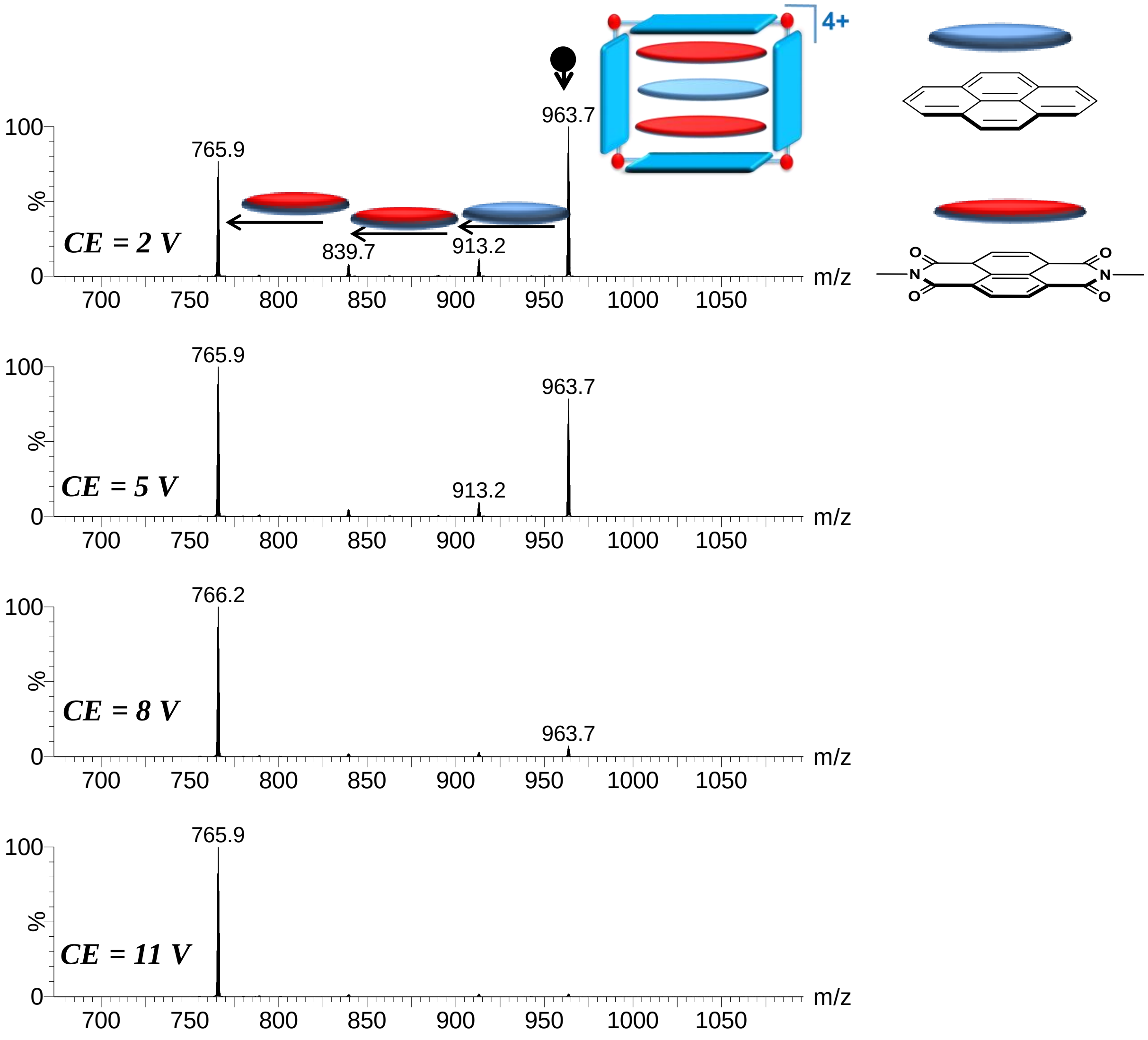

Figure S7. CID mass spectra of the supramolecular $[(NTCDI)_2(pyrene)@\mathbf{1}]^{4+}$ recorded at increasing collision energies from 2 to 11 V from the top to the bottom.

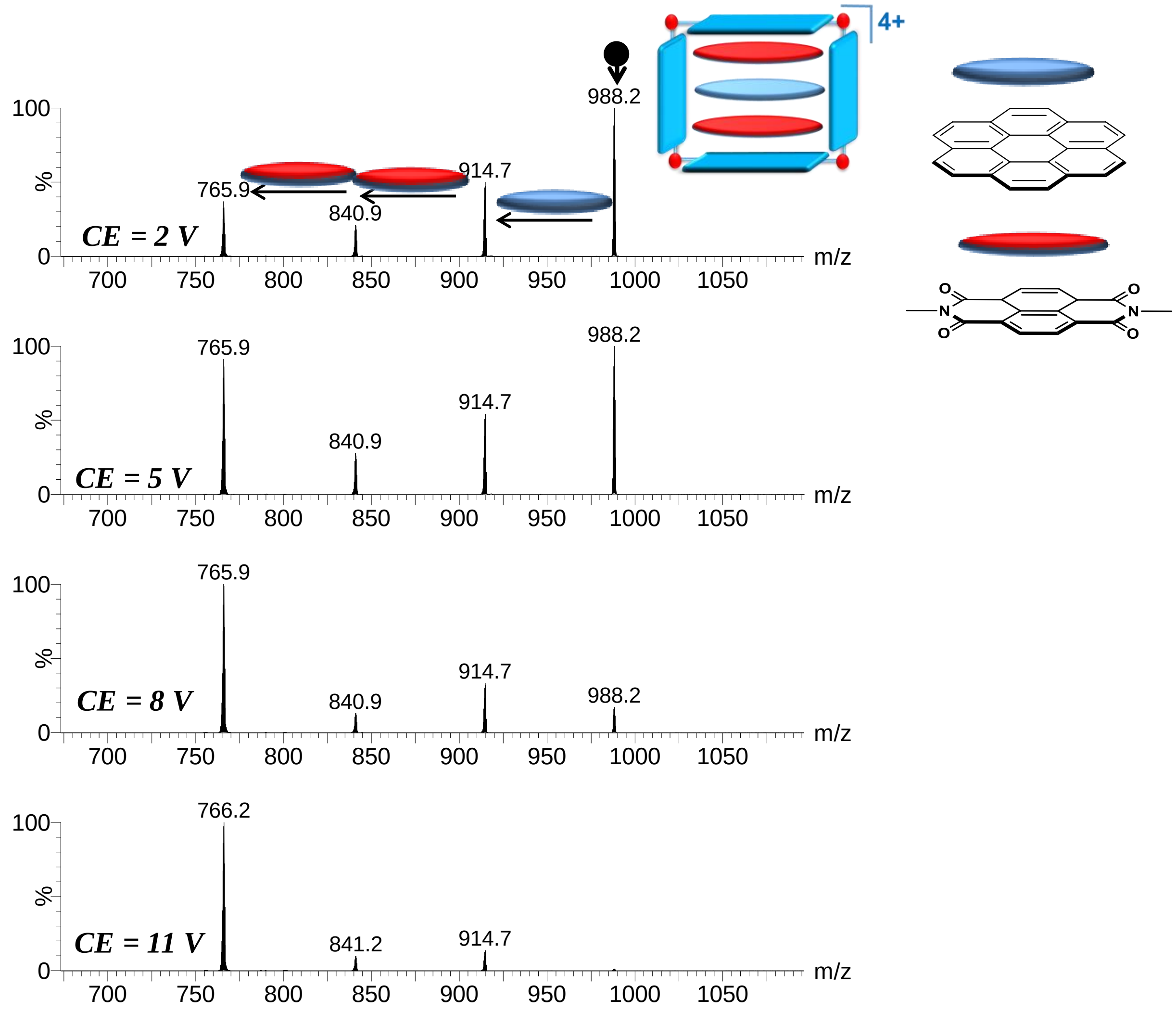

Figure S8. CID mass spectra of the supramolecular [(NTCDI)$_2$(coronene)@**1**]$^{4+}$ recorded at increasing collision energies from 2 to 11 V from the top to the bottom.

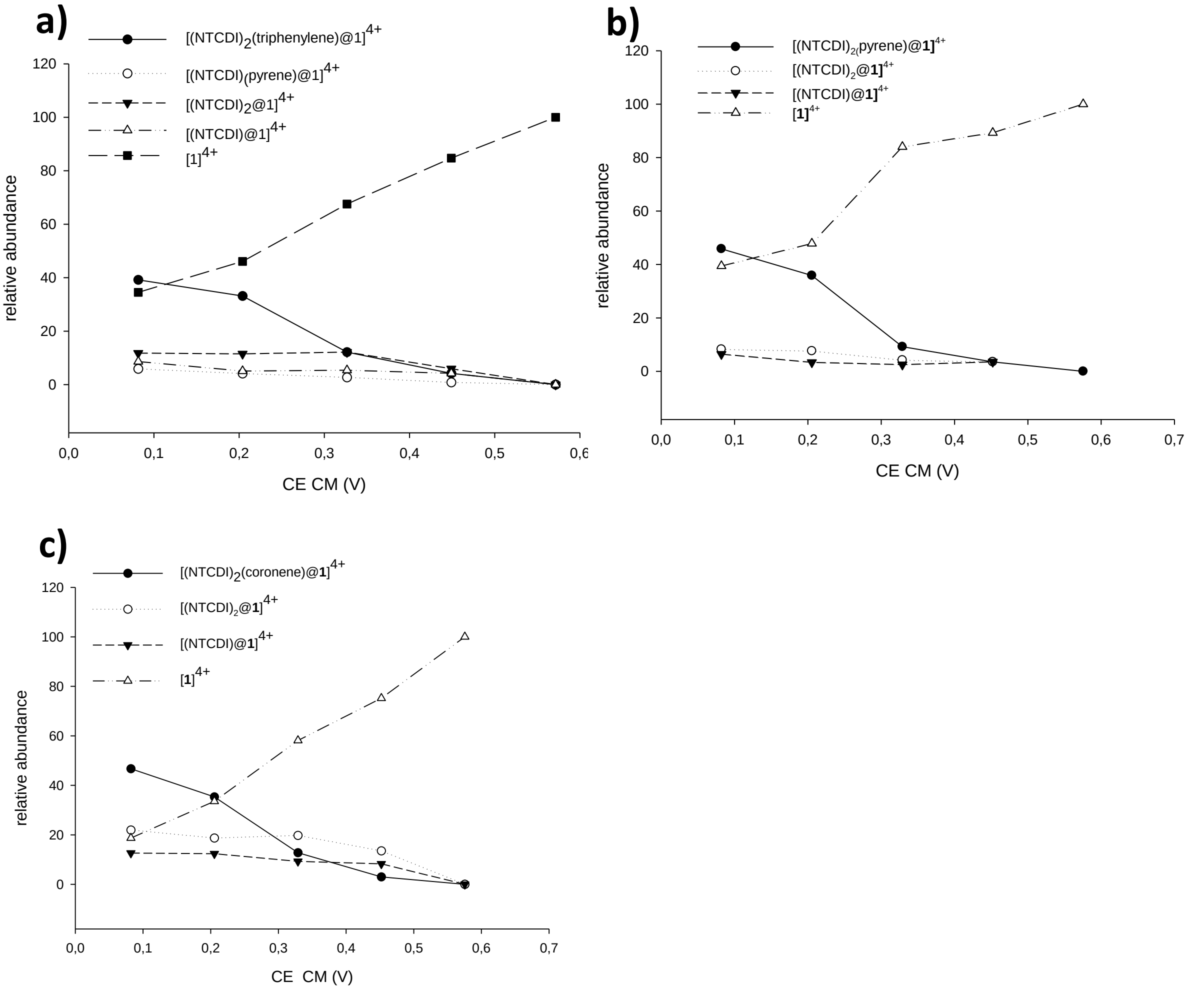

Figure S9-S11. Breakdown profiles for mass-selected [(NTCDI)$_2$(triphenylene)@**1**]$^{4+}$ (a), [(NTCDI)$_2$(pyrene)@**1**]$^{4+}$ (b) and [(NTCDI)$_2$(coronene)@**1**]$^{4+}$ (c). An inspection of the ion abundances of the product ions [**1**]$^{4+}$,[(NTCDI)$_2$@**1**]$^{4+}$ and [(NTCDI)@**1**]$^{4+}$ as the collision energy is raised indicates that the encapsulated guests are expelled in a simultaneous mode. Collision energies (Elab) were converted to the center-of-mass frame, CE CM = q**m*/(*m*+*M*)*E*lab, where q stands for the charge state and *m* and *M* stand for the masses of the collision gas and the ionic species, respectively. For the breakdown profiles representations, signal intensities were obtained from the average of 20 scans and measuring the area of the fragmentation peaks. These graphs were represented taking into account the relative abundance of the precursor and product peaks of each compound (*I*precursor ion or *I*product ion / *I*precursor ion + *I* product ion) against CE CM.

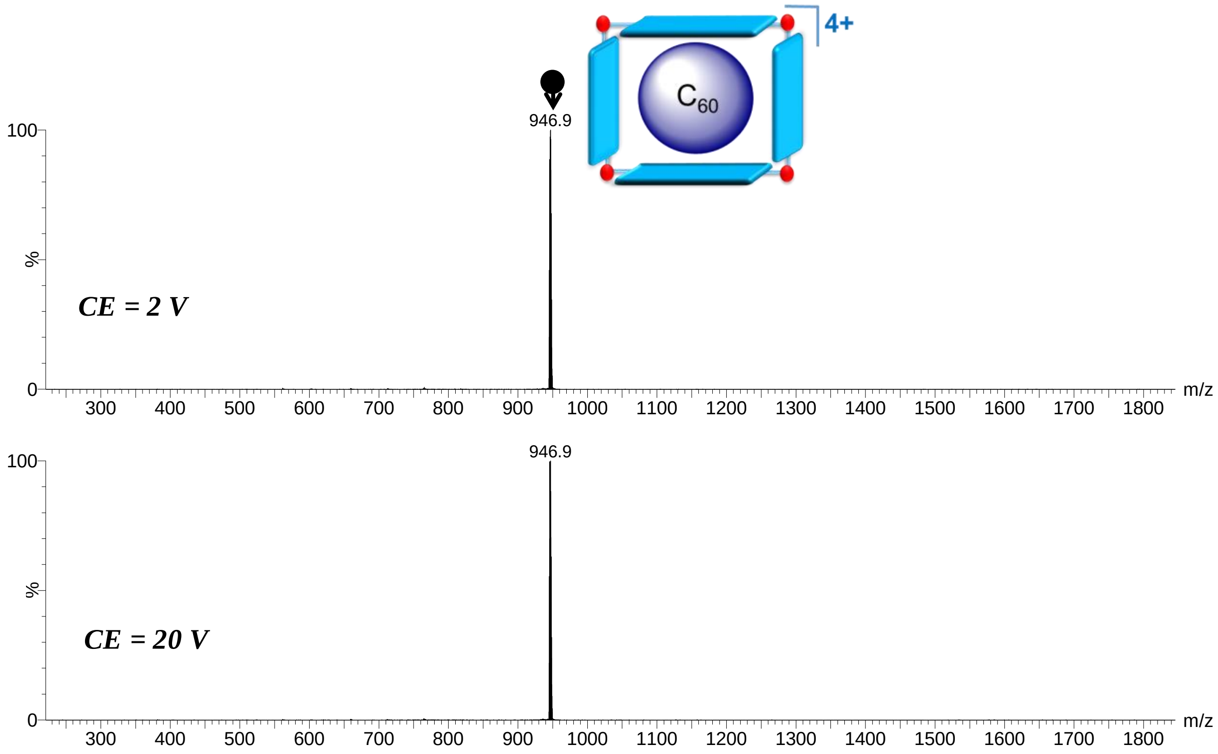

Figure S12. CID mass spectra of the supramolecular $[C_{60}@\mathbf{1}]^{4+}$ recorded at 2 V (top) and 20 V (bottom).

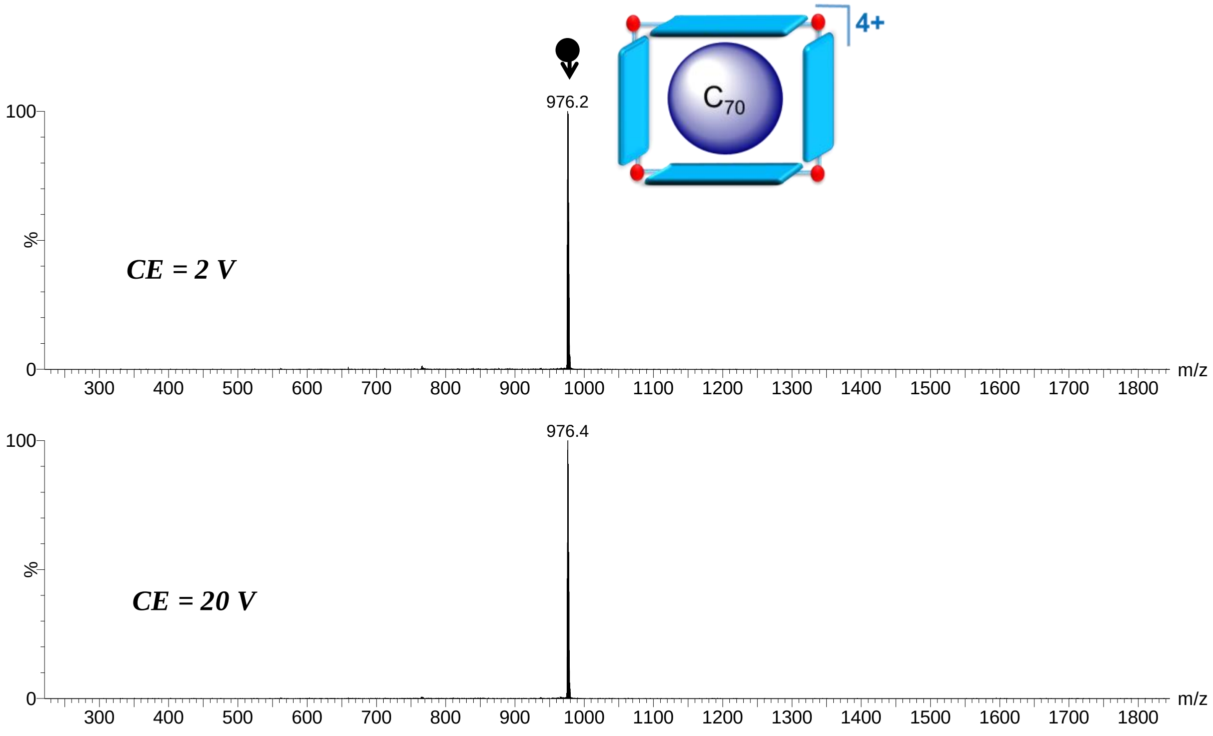

Figure S13. CID mass spectra of the supramolecular $[C_{70}@\mathbf{1}]^{4+}$ recorded at 2 V (top) and 20 V (bottom).

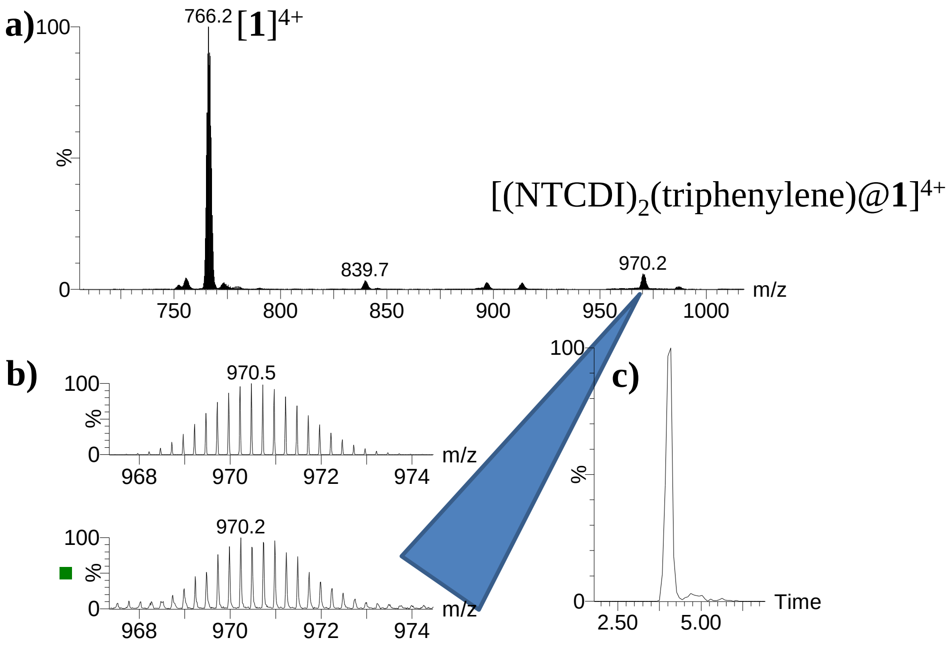

Figure S14. a) TWIM mass spectrum of acetonitrile 1μM solution of compound [(NTCDI]$_2$(triphenylene)@**1**]$^{4+}$; b) comparison between the simulated and experimental isotopic distribution for the [(NTCDI]$_2$(triphenylene)@**1**]$^{4+}$ cation (molecular composition $C_{198}H_{264}N_{16}Pd_4$).; c) arrival time distribution of the [(NTCDI]$_2$(triphenylene)@**1**]$^{4+}$ species.

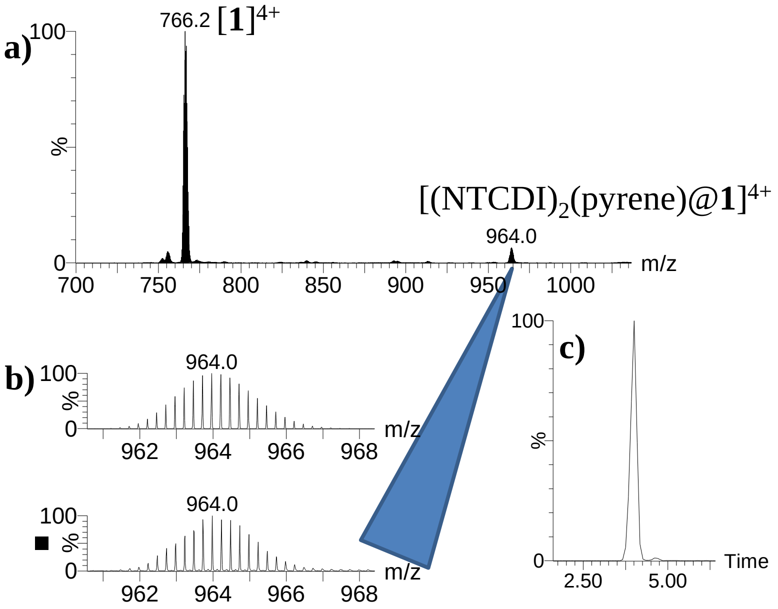

Figure S15. a) TWIM mass spectrum of acetonitrile 1μM solution of compound [(NTCDI]$_2$(pyrene)@**1**]$^{4+}$; b) comparison between the simulated and experimental isotopic distribution for the [(NTCDI]$_2$(pyrene)@**1**]$^{4+}$ cation (molecular composition $C_{196}H_{262}N_{16}Pd_4$); c) arrival time distribution of the [(NTCDI]$_2$(pyrene)@**1**]$^{4+}$ species.

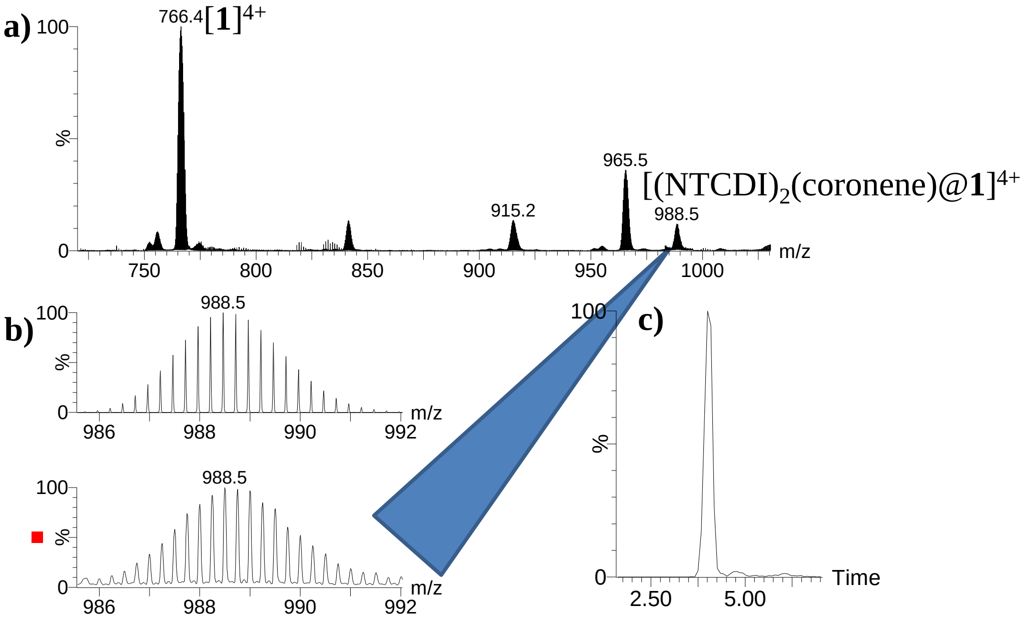

Figure S16. a) TWIM mass spectrum of acetonitrile 1μM solution of compound $[(NTCDI]_2(coronene)@\mathbf{1}]^{4+}$; b) comparison between the simulated and experimental isotopic distribution for the $[(NTCDI]_2(coronene)@\mathbf{1}]^{4+}$ cation (molecular composition $C_{204}H_{264}N_{16}Pd_4$); c) arrival time distribution of the $[(NTCDI]_2(coronene)@\mathbf{1}]^{4+}$ species.

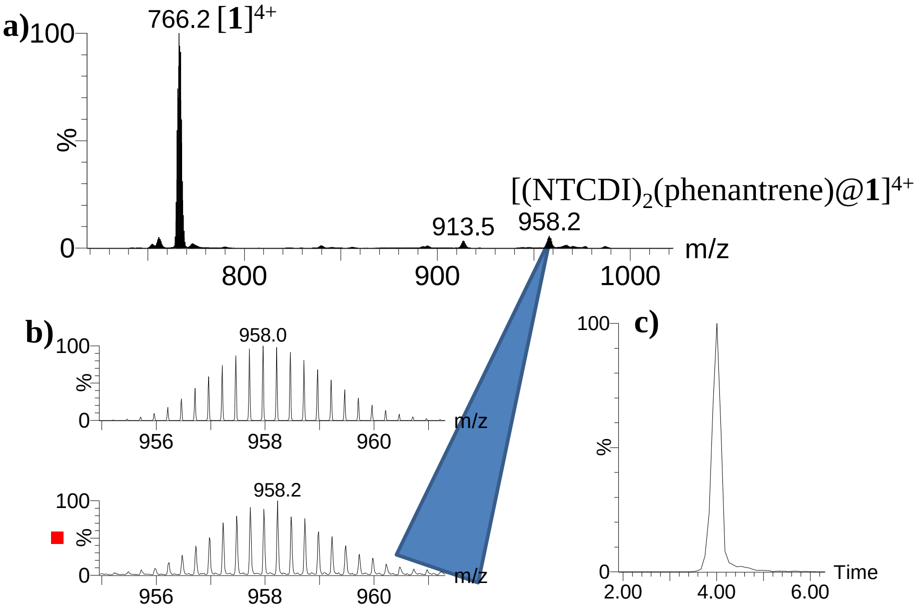

Figure S17. a) TWIM mass spectrum of acetonitrile 1μM solution of compound $[(NTCDI]_2(phenanthrene)@\mathbf{1}]^{4+}$; b) comparison between the simulated and experimental isotopic distribution for the $[(NTCDI]_2(phenanthrene)@\mathbf{1}]^{4+}$ cation (molecular composition $C_{194}H_{262}N_{16}Pd_4$); c) arrival time distribution of the $[(NTCDI]_2(phenanthrene)@\mathbf{1}]^{4+}$ species.

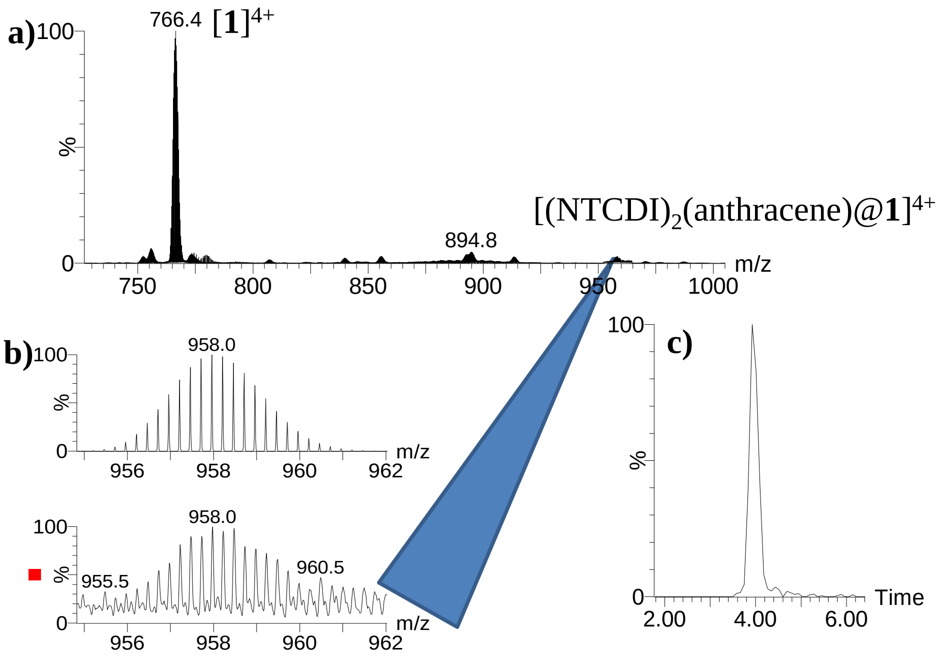

Figure S18. a) TWIM mass spectrum of acetonitrile 1μM solution of compound [(NTCDI]$_2$(anthracene)@**1**]$^{4+}$; b) comparison between the simulated and experimental isotopic distribution for the [(NTCDI]$_2$(anthracene)@**1**]$^{4+}$ cation (molecular composition $C_{194}H_{262}N_{16}Pd_4$); c) arrival time distribution of the [(NTCDI]$_2$(anthracene)@**1**]$^{4+}$ species.

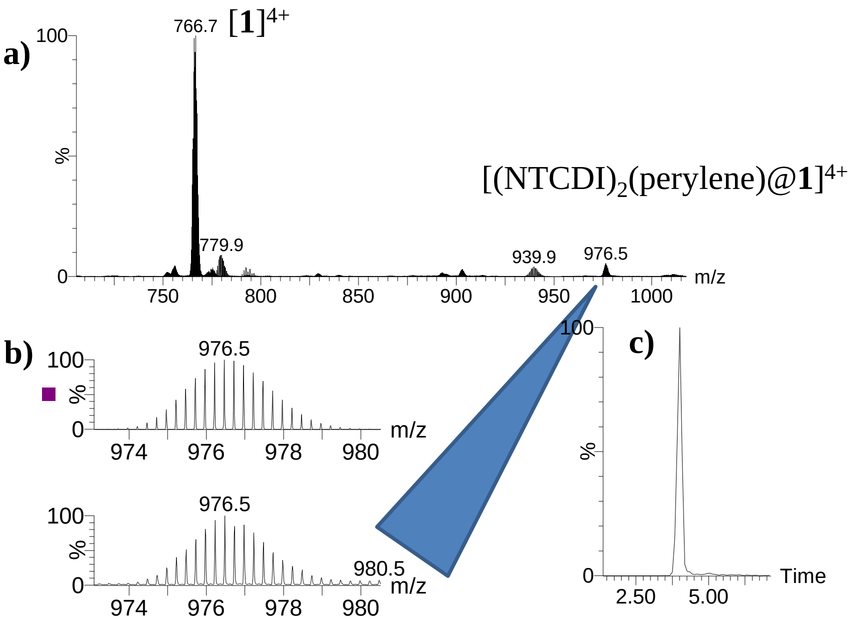

Figure S19. a) TWIM mass spectrum of acetonitrile 1μM solution of compound [(NTCDI]$_2$(perylene)@**1**]$^{4+}$; b) comparison between the simulated and experimental isotopic distribution for the [(NTCDI]$_2$(perylene)@**1**]$^{4+}$ cation (molecular composition $C_{200}H_{264}N_{16}Pd_4$); c) arrival time distribution of the [(NTCDI]$_2$(perylene)@**1**]$^{4+}$ species.

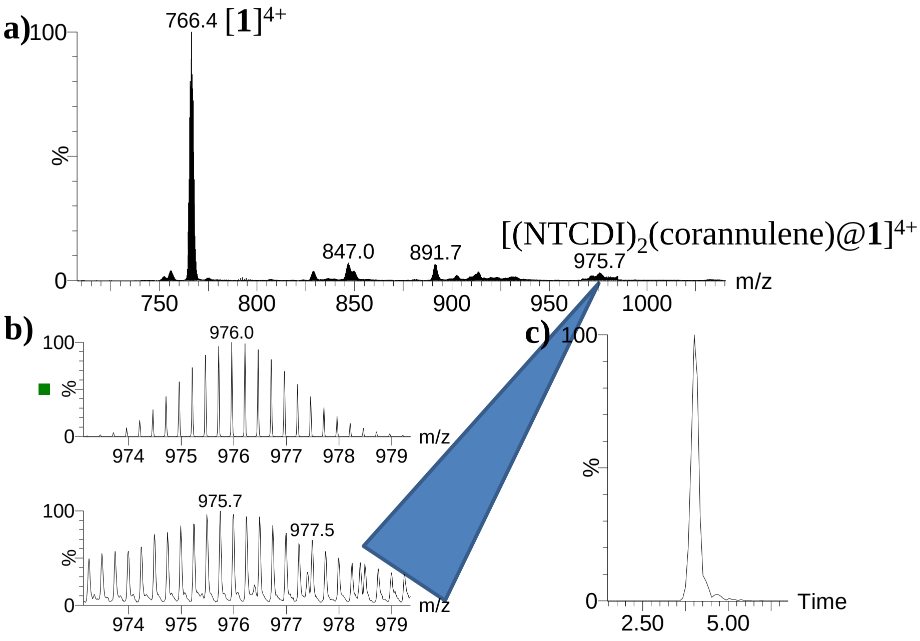

Figure S20. a) TWIM mass spectrum of acetonitrile 1μM solution of compound [(NTCDI]$_2$(corannulene)@**1**]$^{4+}$; b) comparison between the simulated and experimental isotopic distribution for the [(NTCDI]$_2$(corannulene)@**1**]$^{4+}$ cation (molecular composition $C_{200}H_{262}N_{16}Pd_4$); c) arrival time distribution of the [(NTCDI]$_2$(corannulene)@**1**]$^{4+}$ species.

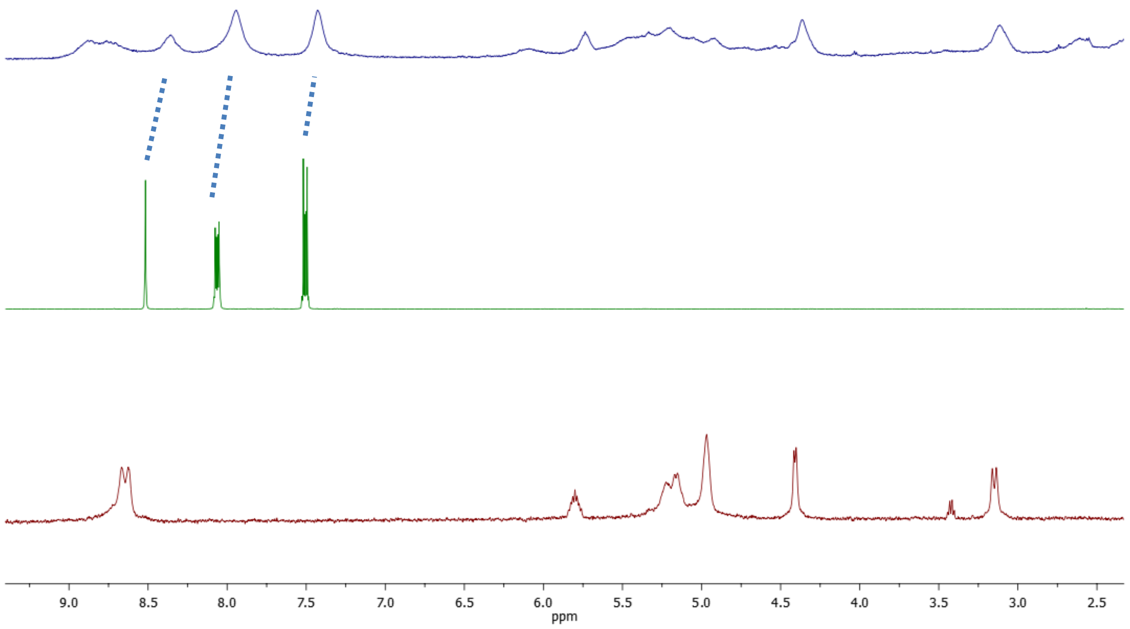

Figure S21. Selected region of the ^{1}H NMR (500 MHz) spectra recorded in CD_3CN of [**1**]$(BF_4)_4$ (bottom) (b) anthracene (middle) and [(NTCDI)$_2$(anthracene)@**1**)]$^{4+}$ (top).

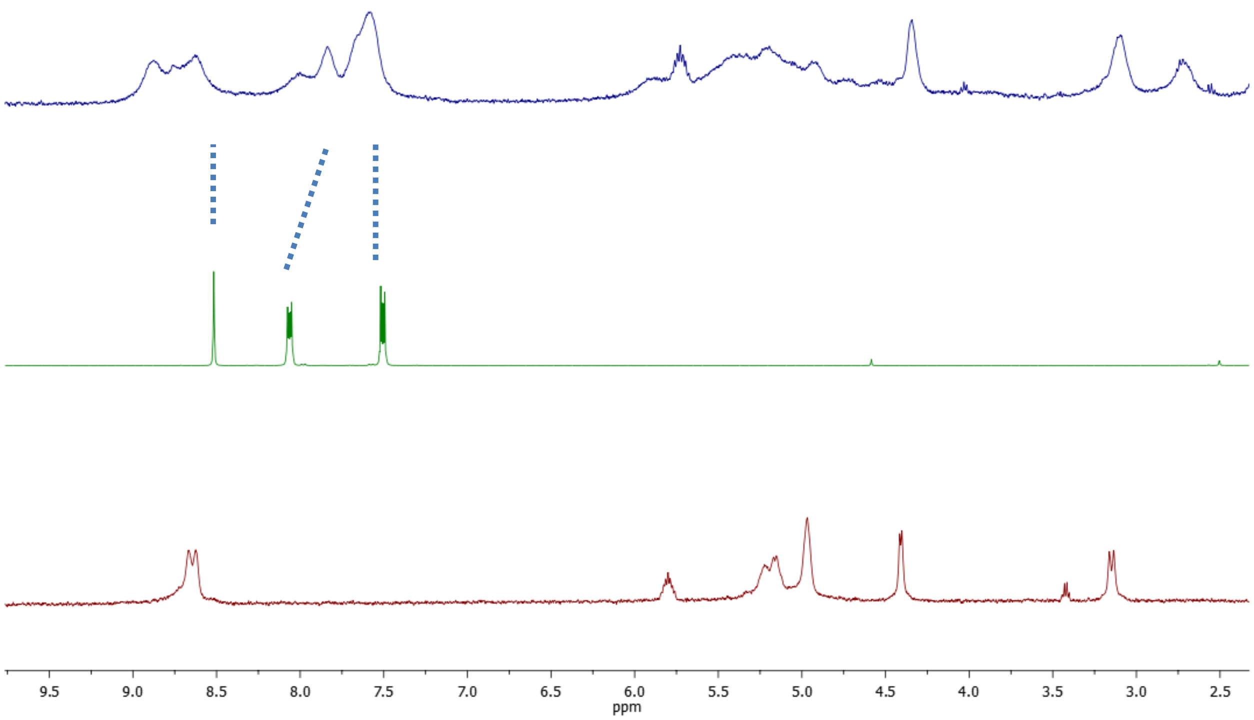

Figure S22. Selected region of the ^{1}H NMR (500 MHz) spectra recorded in CD_3CN of [**1**]$(BF_4)_4$ (bottom) (b) phenenthrene (middle) and $[(NTCDI)_2(phenanthrene)@\mathbf{1})]^{4+}$ (top).

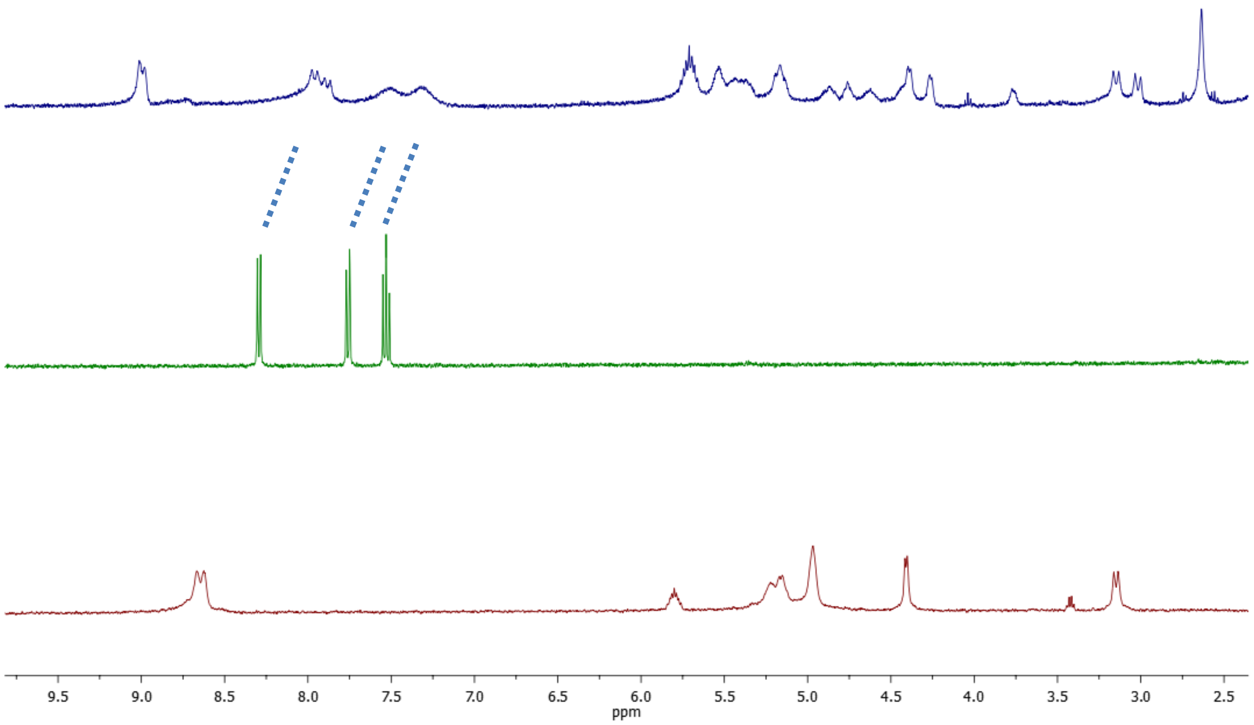

Figure S23. Selected region of the ^{1}H NMR (500 MHz) spectra recorded in CD_3CN of [**1**]$(BF_4)_4$ (bottom) (b) perylene (middle) and $[(NTCDI)_2(perylene)@\mathbf{1})]^{4+}$ (top).

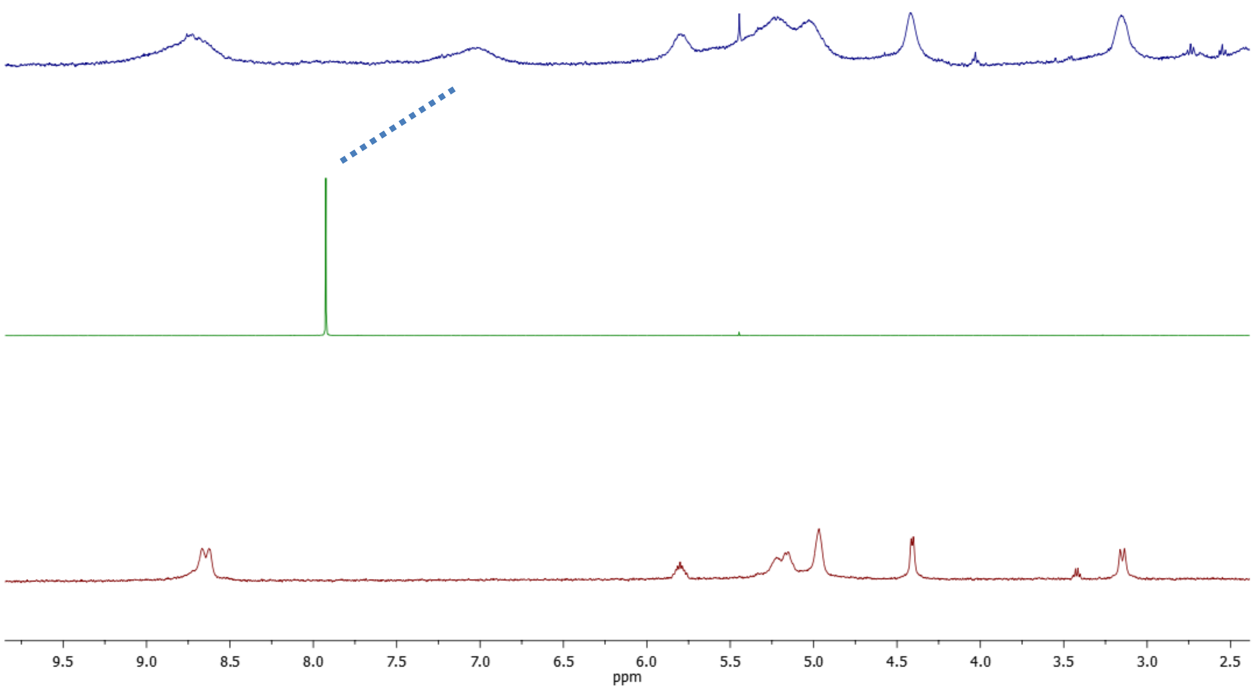

Figure S24. Selected region of the ^{1}H NMR (500 MHz) spectra recorded in CD_3CN of [**1**]$(BF_4)_4$ (bottom) (b) corannulene (middle) and $[(NTCDI)_2(corannulene)@\mathbf{1})]^{4+}$ (top).

3 References

[1] V. Martinez-Agramunt, T. Eder, H. Darmandeh, G. Guisado-Barrios, E. Peris, *Angew. Chem. Int. Ed.* **2019**, *58*, 5682-5686.

[2] V. Martinez-Agramunt, E. Peris, *Chem. Commun.* **2019**, *55*, 14972-14975.

[3] B. T. Ruotolo, J. L. P. Benesch, A. M. Sandercock, S. J. Hyung, C. V. Robinson, *Nat. Protoc.* **2008**, *3*, 1139-1152.

[4] S. M. Stow, T. J. Causon, X. Y. Zheng, R. T. Kurulugama, T. Mairinger, J. C. May, E. E. Rennie, E. S. Baker, R. D. Smith, J. A. McLean, S. Hann, J. C. Fjeldsted, *Analytical Chemistry* **2017**, *89*, 9048-9055.

[5] M. F. Bush, I. D. G. Campuzano, C. V. Robinson, *Anal. Chem.* **2012**, *84*, 7124-7130.

[6] J. Coots, V. Gandhi, T. Onakoya, X. Chen, C. L. Andaluz, *J Aerosol Sci* **2020**, 105570.

[7] Gaussian 16, Revision C.01, M. J. Frisch, G. W. Trucks, H. B. Schlegel, G. E. Scuseria, M. A. Robb, J. R. Cheeseman, G. Scalmani, V. Barone, G. A. Petersson, H. Nakatsuji, X. Li, M. Caricato, A. V. Marenich, J. Bloino, B. G. Janesko, R. Gomperts, B. Mennucci, H. P. Hratchian, J. V. Ortiz, A. F. Izmaylov, J. L. Sonnenberg, D. Williams-Young, F. Ding, F. Lipparini, F. Egidi, J. Goings, B. Peng, A. Petrone, T. Henderson, D. Ranasinghe, V. G. Zakrzewski, J. Gao, N. Rega, G. Zheng, W. Liang, M. Hada, M. Ehara, K. Toyota, R. Fukuda, J. Hasegawa, M. Ishida, T. Nakajima, Y. Honda, O. Kitao, H. Nakai, T. Vreven, K. Throssell, J. A. Montgomery, Jr., J. E. Peralta, F. Ogliaro, M. J. Bearpark, J. J. Heyd, E. N. Brothers, K. N. Kudin, V. N. Staroverov, T. A. Keith, R. Kobayashi, J. Normand, K. Raghavachari, A. P. Rendell, J. C. Burant, S. S. Iyengar, J. Tomasi, M. Cossi, J. M. Millam, M. Klene, C. Adamo, R. Cammi, J. W. Ochterski, R. L. Martin, K. Morokuma, O. Farkas, J. B. Foresman, and D. J. Fox, Gaussian, Inc., Wallingford CT, 2016.

[8], H. S. Yu, H. He, D. G. Truhlar, *J. Chem. Theory Comput.* **2016**, *12*, 1280-1293.

[9] a) F. Weigend, R. Ahlrichs, *Phys. Chem. Chem. Phys.* **2005**, *7*, 3297-3305.; b) F. Weigend, *Phys. Chem. Chem. Phys.* **2006**, *8*, 1057-1065

[10] J. P. Williams, J. H. Scrivens, *Rapid Commun. Mass Spectrom.* **2008**, *22*, 187-196.

4 Cartesian coordinates for the calculated structures

Atom coordinates od [1’]$^{4+}$, [C132H156N16Pd4]

Pd	-9.22830700	2.95320000	-3.23535400
C	-8.69377000	1.83199000	-1.56580900
C	-8.12540800	4.35065600	-2.16170900
C	-10.41793400	1.65219300	-4.53161700
C	-9.86188900	2.71008100	-5.29521300
C	-10.11083300	4.04852500	-4.90085100
N	-7.42701900	1.70664400	-1.04891400
N	-9.54246900	1.71324200	-0.50145300
N	-6.80673900	4.53920700	-2.46515500
N	-8.25151600	4.65617400	-0.82941000
H	-10.11757300	0.60998900	-4.72621400
H	-11.41578200	1.79007000	-4.07347700
H	-9.04009400	2.49270200	-6.00441900
H	-9.57542400	4.88028100	-5.38828200
H	-11.08028000	4.31356100	-4.43885100
C	-7.47781200	1.58442800	0.34341600
C	-6.26575800	1.47886400	-1.91268700
C	-8.82693000	1.57656400	0.69250100
C	-10.99330000	1.64581100	-0.68219300
C	-6.08161000	4.89328400	-1.32274300
C	-6.31742700	4.45627100	-3.84241700
C	-7.00149100	4.95694900	-0.27785000
C	-9.56590100	4.85724100	-0.21362200
C	-6.43083200	1.47186100	1.33012700
H	-5.52035100	2.29834000	-1.85554200
H	-5.77517900	0.52544200	-1.62757200
H	-6.64276000	1.40185400	-2.95166900
C	-9.28090100	1.43654700	2.05648700
H	-11.49416200	2.57882700	-0.34749400
H	-11.18656100	1.49749900	-1.75980400
H	-11.40112700	0.78348000	-0.11871600
C	-4.67479900	5.16109400	-1.14101300
H	-5.70161100	5.34746400	-4.07474600
H	-5.71967600	3.53557100	-4.01182900
H	-7.19971900	4.44059600	-4.50852300
C	-6.60612400	5.26352000	1.07538900
H	-9.78394000	4.11819000	0.58423300
H	-9.62106400	5.87856900	0.21588500
H	-10.32119200	4.75699700	-1.01808100
C	-6.86416400	1.08404400	2.64868000
C	-5.06279400	1.74398000	1.08581600
C	-8.26292600	1.07980100	3.01717400
C	-10.61088800	1.63779600	2.48447700

C	-4.30612900	5.70318600	0.14680200
C	-3.67759200	4.91025400	-2.10848700
C	-5.25727900	5.74696700	1.23519900
C	-7.43307600	5.07531600	2.20869200
C	-5.84073700	0.76066300	3.61075500
C	-4.08344900	1.58105200	2.08507400
H	-4.77122500	2.12986300	0.10123000
C	-8.68756500	0.79160500	4.36772700
C	-11.00538600	1.45373500	3.82517500
H	-11.36801500	1.97773700	1.76749700
C	-2.93832200	6.12851100	0.32322300
C	-2.32095800	5.22503500	-1.89056200
H	-3.94772700	4.42941500	-3.05643200
C	-4.86974400	6.21177300	2.54556600
C	-6.99515400	5.40024200	3.50562100
H	-8.42521400	4.62923400	2.07255000
C	-6.32615900	0.16440600	4.83204300
C	-4.48874600	1.05522800	3.33019300
C	-2.60154400	1.95441100	1.86718700
C	-7.65827200	0.24402300	5.22870500
C	-10.03726700	1.00151300	4.74111300
C	-12.46612500	1.74551600	4.22289900
C	-2.66547700	6.81767100	1.56559300
C	-1.98016800	5.86667300	-0.68624500
C	-1.28357200	4.86223200	-2.97172200
C	-3.58596600	6.87010100	2.60972100
C	-5.72390800	5.99375600	3.64657000
C	-7.83196100	5.11152200	4.76830100
N	-5.67011700	-0.62137000	5.78474300
H	-3.73611000	0.91658400	4.12207300
C	-2.31271600	2.33455700	0.40234400
C	-2.26838200	3.17795800	2.75776900
C	-1.68233400	0.77001500	2.24829100
N	-7.73879100	-0.44292800	6.44695200
H	-10.34228800	0.83339100	5.77756300
C	-13.40503500	0.79657100	3.43913400
C	-12.80864400	3.21412300	3.87162100
C	-12.71178500	1.54475700	5.72998200
N	-1.58631400	7.61883200	1.95585600
H	-0.93329100	6.13295900	-0.51592300
C	-1.61611800	5.62889300	-4.27454500
C	0.15245400	5.21907900	-2.54460000
C	-1.33938300	3.33855100	-3.24143700
N	-3.03335000	7.71136700	3.58344500
H	-5.37668900	6.25380200	4.65501600
C	-7.09791400	4.01265600	5.57548500

C	-7.97915100	6.38737200	5.62950800
C	-9.24196700	4.59895900	4.42448200
C	-6.53924600	-1.02123200	6.76345700
C	-4.30940500	-1.14656400	5.66945000
H	-2.52625600	1.49200900	-0.29128300
H	-2.90640500	3.21482800	0.08550900
H	-1.23893600	2.60176600	0.29224300
H	-2.96163900	4.01958600	2.54057700
H	-2.34908700	2.93068600	3.84191400
H	-1.22515700	3.51495700	2.55323200
H	-1.73373400	0.52412400	3.33208700
H	-1.94163700	-0.14065200	1.66777800
H	-0.62591900	1.03234600	2.02241300
C	-8.90998500	-0.52676300	7.31797800
H	-13.32041000	0.94299900	2.34101200
H	-13.17958600	-0.26718800	3.66449600
H	-14.46051800	0.99478200	3.72200100
H	-12.15056900	3.92192700	4.42025000
H	-12.71222800	3.41978700	2.78429100
H	-13.85992600	3.43044400	4.15700000
H	-12.53555800	0.49305100	6.04274800
H	-12.06869300	2.21490600	6.34209500
H	-13.76801800	1.78699100	5.96617400
C	-1.82138100	8.18936500	3.17607000
C	-0.36759000	7.87348200	1.19270300
H	-2.62066200	5.36234200	-4.66717500
H	-1.58923100	6.72696400	-4.11155500
H	-0.87245600	5.37654000	-5.05986700
H	0.27855800	6.31129500	-2.38365600
H	0.44736500	4.68221900	-1.61583600
H	0.85979000	4.91912500	-3.34453800
H	-2.33265000	3.01457000	-3.61853600
H	-0.58820600	3.06755000	-4.01334100
H	-1.11107300	2.76050000	-2.31969900
C	-3.67475000	8.17253400	4.81333300
H	-6.92443900	3.11696200	4.94120600
H	-6.11163400	4.36993900	5.94446600
H	-7.70986300	3.71204100	6.45580800
H	-7.00262600	6.77100400	5.99336400
H	-8.48007200	7.19832000	5.05996800
H	-8.59903200	6.16372200	6.52386900
H	-9.82371500	5.34740100	3.84295500
H	-9.20051800	3.65363100	3.84854100
H	-9.79937300	4.39095000	5.36381100
Pd	-6.20126600	-2.38775800	8.28854400
H	-3.56805400	-0.47494800	6.16061700

H	-4.28217200	-2.13841000	6.15715300
H	-4.05247800	-1.26972400	4.60017400
H	-8.61070600	-1.06187200	8.23753000
H	-9.25106300	0.49058900	7.59483500
H	-9.73799000	-1.07334400	6.82198400
Pd	-0.54579800	9.43214500	4.21524300
H	0.23312700	6.94647200	1.08755100
H	-0.61146000	8.27179800	0.18746200
H	0.22751700	8.61894600	1.75015500
H	-4.72497900	8.45965300	4.60767000
H	-3.64908600	7.38743500	5.59834700
H	-3.11670700	9.05920700	5.16969600
C	-4.39879000	-1.55987400	8.85291100
C	-7.59955700	-3.75374200	9.23750800
C	-6.28700500	-4.06607600	9.68376200
C	-7.86489700	-3.74802300	7.84807900
C	-0.24622200	8.09147200	5.77067200
C	-0.47148800	11.12367800	2.83488700
C	0.95905700	10.91618700	4.80237900
C	-0.11131800	11.55997700	4.13848300
N	-3.16794400	-2.14688700	8.75159800
N	-4.17343000	-0.30376600	9.33723400
H	-8.31032700	-3.26475500	9.93204000
H	-5.71859500	-4.84701800	9.14503800
H	-6.02111300	-3.93317300	10.74518100
H	-7.32428300	-4.45078500	7.18660900
H	-8.83677400	-3.39395100	7.46752100
N	0.06310400	6.76099100	5.68611100
N	-0.17347400	8.40073300	7.10213200
H	0.33301700	10.82172100	2.13858400
H	-1.38965600	11.50742100	2.36087900
H	1.80846400	10.52248000	4.21317100
H	1.18307900	11.14999700	5.85587800
H	-0.81606400	12.18771800	4.71811000
C	-2.16660500	-1.28522900	9.21389300
C	-2.98666500	-3.50675600	8.25103300
C	-2.80858200	-0.10440500	9.57996700
C	-5.26787000	0.60490900	9.67373300
C	0.37512500	6.24510300	6.94774100
C	0.24866200	6.06877200	4.41051200
C	0.26815600	7.30075900	7.84890000
C	-0.55543100	9.70891500	7.63127500
C	-0.75143000	-1.49831500	9.42558600
H	-2.41448500	-3.50447200	7.30031000
H	-2.45845000	-4.13179600	8.99867300
H	-3.98905600	-3.92994900	8.05923000

C	-2.10455700	1.01145600	10.16747800
H	-5.11454300	1.02252800	10.68853800
H	-5.34339400	1.43239800	8.93704800
H	-6.20645100	0.01889900	9.65801500
C	0.80634200	4.92558300	7.34206800
H	-0.68193900	5.54727700	4.08831100
H	0.52793000	6.82066200	3.64952800
H	1.07355700	5.33736300	4.50620000
C	0.73642400	7.19670300	9.21664400
H	-0.94276000	10.31425100	6.79155500
H	-1.35911300	9.59346300	8.38545300
H	0.31342800	10.22273100	8.09111100
C	-0.09983700	-0.50715300	10.24767400
C	-0.00621800	-2.58112900	8.89838500
C	-0.76312800	0.72399500	10.61592600
C	-2.62606100	2.31446700	10.30983200
C	1.49481500	4.86890500	8.60704100
C	0.57965800	3.74180700	6.60695000
C	1.47620000	5.99265100	9.51745700
C	0.56432800	8.18510600	10.21568100
C	1.27139300	-0.75521900	10.63025800
C	1.36248700	-2.75011000	9.17471500
H	-0.49299200	-3.29032000	8.22317300
C	-0.08803000	1.74122200	11.38257300
C	-1.90776100	3.34606100	10.94877400
H	-3.60476300	2.55158600	9.87290600
C	2.14414700	3.62892600	8.94922000
C	1.09037900	2.49508700	7.02645800
H	-0.05328400	3.78474600	5.70674200
C	2.14210100	5.91726800	10.79709300
C	1.12510900	8.05612800	11.50013200
H	-0.04287800	9.06876800	10.00075500
C	1.83300300	0.18131200	11.57438800
C	1.97145700	-1.84369800	10.06619900
C	2.21168400	-3.87709200	8.55361400
C	1.18388600	1.35755600	11.94461300
C	-0.65570200	3.03154800	11.50816400
C	-2.50551100	4.76705600	10.98194200
C	2.99171000	3.67609700	10.11609900
C	1.90343900	2.46694200	8.17647000
C	0.74584000	1.22508800	6.21920100
C	2.98186800	4.75858100	10.99382400
C	1.93248400	6.92985900	11.75817800
C	0.90093000	9.09074300	12.62108400
N	3.03928000	0.15732200	12.28178900
H	3.03383000	-1.98619600	10.29806900

C	3.40593700	-3.25401000	7.79011800
C	1.40519400	-4.73483100	7.56082100
C	2.74329100	-4.79511700	9.68067900
N	2.01283200	1.99380100	12.87485900
H	-0.08482200	3.81329200	12.02288800
C	-3.94419000	4.74236800	11.54833800
C	-2.51998300	5.30185200	9.52889000
C	-1.66738800	5.72805800	11.84408500
N	3.93476600	2.76977100	10.61165000
H	2.33317400	1.51487600	8.51123400
C	-0.76519200	0.92715000	6.39096700
C	1.53634100	-0.00233900	6.71128200
C	1.06464400	1.43351400	4.71977100
N	3.90724000	4.45831000	11.99851000
H	2.38087400	6.83621500	12.75471300
C	0.28899500	8.38523700	13.85630600
C	2.26106500	9.71949100	13.00959500
C	-0.05739900	10.21728800	12.19179800
C	3.17461900	1.28240400	13.04482500
C	4.02408800	-0.92485600	12.32959900
H	4.07395500	-2.67094500	8.45912500
H	4.01819500	-4.05935100	7.33172500
H	3.05443700	-2.58235200	6.97699800
H	0.55735300	-5.25563800	8.05544500
H	1.01015500	-4.12298300	6.71979800
H	2.06452700	-5.51448200	7.12764600
H	3.38857300	-4.24177900	10.39603300
H	1.90860300	-5.25368600	10.25169000
H	3.35438900	-5.61267400	9.24296500
C	1.66378600	3.10683300	13.76188500
H	-4.63706200	4.13726600	10.92632400
H	-3.96011500	4.33563400	12.58141700
H	-4.34924500	5.77628800	11.58128800
H	-1.50112400	5.25188600	9.08853100
H	-3.20355900	4.70709100	8.88422200
H	-2.86257900	6.36123900	9.51449300
H	-1.62018500	5.39690300	12.90468500
H	-0.63480700	5.82731700	11.45555300
H	-2.12762200	6.74010100	11.82421000
C	4.46559200	3.22817700	11.79311600
C	4.51249400	1.62343500	9.90555200
H	-1.02682400	0.83160300	7.46739800
H	-1.39524300	1.73400800	5.94929000
H	-1.01601400	-0.03110400	5.87859200
H	2.63319600	0.14384900	6.60113300
H	1.31381600	-0.23253100	7.77204300

H	1.25009200	-0.89370900	6.11124100
H	0.42748500	2.21822800	4.25503200
H	2.12979700	1.71134600	4.57207200
H	0.87592800	0.48858300	4.16535100
C	4.34723900	5.32698300	13.09120500
H	-0.68940900	7.91953200	13.60934900
H	0.95698800	7.59759800	14.26516600
H	0.12176800	9.12808100	14.66477900
H	2.97551700	8.95829800	13.38998900
H	2.72958600	10.23012600	12.14187900
H	2.11195100	10.46990900	13.81459200
H	0.34845800	10.80661200	11.34157600
H	-1.05638900	9.81811100	11.90864200
H	-0.20434800	10.91812600	13.03874700
Pd	5.07247200	2.05230800	13.39913000
H	3.50732000	-1.89443700	12.47203800
H	4.63918300	-0.96251500	11.40548700
H	4.68537500	-0.73502600	13.19508900
H	1.58854900	4.07570000	13.22719600
H	0.69568000	2.89369400	14.25985700
H	2.46208400	3.17564400	14.52718200
H	3.80552300	0.77336700	9.81548400
H	4.82939100	1.93725200	8.88964000
H	5.40034900	1.29875000	10.48333800
H	3.58803100	5.38931600	13.89943700
H	5.27900800	4.89785100	13.50138400
H	4.55716600	6.34228500	12.70075600
C	5.75496600	1.11813900	15.24688500
C	7.08853500	2.70214700	13.94875400
C	6.86918400	1.37933900	14.41066300
H	5.48127200	0.08034800	15.50014300
H	5.41525000	1.88776900	15.96508800
H	7.85374700	2.90235100	13.18152200
H	6.83858500	3.54971000	14.61490300
H	7.38777900	0.53804200	13.91182500

Atom coordinates od [C_{60}@1']$^{4+}$, [C192H156N16Pd4]

Pd	28.96067700	4.23180900	14.92152000
C	28.24216200	6.17242100	14.93092200
C	27.16498500	3.21240100	14.90393000
C	31.07134600	4.75747700	15.13290300
C	30.95622800	3.50631600	14.47502700
C	30.21983500	2.45978900	15.08812300
N	27.93214400	6.93128100	13.83847800
N	28.07567900	6.99048900	16.01090700
N	26.43259000	2.83556600	15.99443200
N	26.51240000	2.69685600	13.82105000
H	31.14791400	4.78501000	16.23677700
H	31.51158900	5.61855000	14.60433500
H	31.19538600	3.43997900	13.39607700
H	30.23892900	2.35111700	16.18927100
H	30.00165900	1.53702100	14.52549500
C	27.58742100	8.23444500	14.21603400
C	27.88769500	6.34653900	12.50333600
C	27.64599200	8.26110900	15.61005000
C	28.40781100	6.55171900	17.36323200
C	26.86673900	3.15089800	17.35205800
C	25.32350300	2.07114800	15.61048900
C	27.07358200	2.75626200	12.47360400
C	25.36353000	2.00495700	14.21830000
C	27.12014100	9.35321400	13.43329600
H	28.22951700	5.30034400	12.59900600
H	28.56712000	6.87575300	11.80700500
H	26.84586500	6.36688200	12.10765500
C	27.17972500	9.38488900	16.38205600
H	28.99898600	7.32991900	17.88326300
H	27.48937300	6.32339700	17.94779400
H	29.02597600	5.63832700	17.27582000
H	26.88573800	2.23448600	17.97377200
H	26.19949000	3.90898100	17.81865200
H	27.89453800	3.55462200	17.28569100
C	24.24474500	1.50128400	16.38127900
H	26.92930800	1.78289300	11.96651000
H	26.59913300	3.56343700	11.87363300
H	28.15904600	2.95504300	12.56723100
C	24.27460300	1.47687000	13.43728600
C	26.72905400	10.52444300	14.17748500
C	26.94283700	9.31503000	12.02792900
C	26.77491700	10.54236800	15.62408600
C	26.97492100	9.35005200	17.78408100
C	24.06361900	1.69872800	17.77243300
C	23.26083300	0.76308800	15.62791700

C	24.10063800	1.74074100	12.05586400
C	23.24698500	0.79083900	14.18048800
C	26.16788700	11.63231600	13.43172800
C	26.31205600	10.35077200	11.32584900
H	27.26637900	8.43667600	11.46886600
C	26.29074800	11.68119900	16.36944900
C	26.38326700	10.41342900	18.48281200
H	27.21949300	8.44127600	18.33776700
C	22.96716100	1.16850300	18.47080700
H	24.76494800	2.33508400	18.31685700
C	22.19729400	0.12954700	16.37125500
C	22.95543200	1.32506000	11.35936500
H	24.83666000	2.35774700	11.53228600
C	22.11919200	0.27539800	13.43558300
C	25.86033400	12.81478500	14.20415300
C	25.93463600	11.50075100	12.04747600
C	25.96886900	10.24828700	9.82612800
C	25.97787800	12.85189400	15.58790700
C	26.08076900	11.58562200	17.75914400
C	26.04321000	10.33681700	19.98489600
C	22.70034800	1.47342200	19.95914500
C	22.07369200	0.34328800	17.75886600
C	21.23558200	-0.60417200	15.58559300
C	22.69791300	1.67570700	9.87931800
C	22.00311600	0.55711100	12.05858600
C	21.15624700	-0.48070000	14.20366900
N	25.49125900	14.10823000	13.81313100
H	25.42142700	12.30044700	11.50251500
C	24.42658500	10.24385900	9.68052000
C	26.56642800	11.45180800	9.06048600
C	26.50201200	8.94872000	9.19314200
N	25.72230100	14.17222000	15.98259200
H	25.62605200	12.43188300	18.29174500
C	26.45292300	8.98955300	20.60925800
C	26.77247900	11.47438700	20.73872100
C	24.51270100	10.49089200	20.16204900
C	23.73664900	2.44831200	20.55014400
C	22.73811500	0.15481200	20.76725000
C	21.30368800	2.12800400	20.10450500
H	21.21852400	-0.08972600	18.29466200
N	20.19712800	-1.46066600	15.97570200
C	23.73274200	2.67712800	9.33069100
C	22.76843500	0.37657700	9.04114000
C	21.28951100	2.30395900	9.72025600
H	21.12660900	0.20744000	11.50419700
N	20.04601200	-1.23720800	13.81038000

C	25.41858000	14.94197700	14.89185100
C	25.27898300	14.59045700	12.45326400
H	23.95723900	11.13178000	10.15818800
H	23.99830300	9.33324800	10.15378000
H	24.14602000	10.24123700	8.60460100
H	26.33201400	11.36397800	7.97846100
H	27.67034000	11.48990200	9.17310900
H	26.15009400	12.41987700	9.41182800
H	26.22928100	8.92342400	8.11803600
H	27.60865400	8.87678600	9.26118400
H	26.05161800	8.05068500	9.67426800
C	26.07808700	14.76672700	17.27534500
H	27.54857900	8.81683800	20.54491000
H	25.92394400	8.14069900	20.11920400
H	26.17556400	8.98209800	21.68349800
H	26.54858200	11.41133900	21.82478100
H	26.45003200	12.47836700	20.38955300
H	27.87253500	11.40061700	20.60704000
H	24.12628500	11.41459800	19.67972800
H	23.98022000	9.62168100	19.71788300
H	24.25905400	10.53560100	21.24351900
H	24.76439500	2.02698900	20.52664900
H	23.73284900	3.42123200	20.00747200
H	23.48574300	2.65285200	21.61137300
H	22.55197300	0.36308100	21.84238500
H	21.96145800	-0.56144500	20.42399400
H	23.72715600	-0.34143900	20.67612600
H	21.08723100	2.31702500	21.17838600
H	21.26143800	3.10150600	19.56771900
H	20.49360500	1.47828700	19.70871500
C	20.08228100	-2.14850800	17.26564200
C	19.46222700	-1.84173700	14.88583800
H	24.75974700	2.25350400	9.33261200
H	23.73480200	3.62439500	9.91602100
H	23.48205100	2.92667300	8.27909800
H	22.00664300	-0.36227200	9.37099600
H	22.58060600	0.60342500	7.96981600
H	23.76762700	-0.10011500	9.12595900
H	20.48207100	1.59709100	10.00610800
H	21.17642600	3.22455500	10.33378100
H	21.12277700	2.57625200	8.65555000
C	19.57147300	-1.46274200	12.45029500
Pd	24.64171200	16.85571000	4.85554400
H	26.16721700	14.37932800	11.82415000
H	24.38329200	14.11438600	12.00296700
H	25.12520300	15.68618600	12.50706200

H	27.05526400	14.36123900	17.60720300
H	25.32216300	14.56662100	18.05915400
H	26.16431600	15.86085700	17.12663800
H	19.46009700	-3.05120100	17.10848700
H	21.09177400	-2.45172200	17.60910200
H	19.61199200	-1.51691700	18.04423100
Pd	17.61793100	-2.76661100	14.85545600
H	20.38686200	-1.86895600	11.81844900
H	19.19140300	-0.52007500	12.00602400
H	18.74983900	-2.20381000	12.50067900
C	22.79145900	15.93907100	14.82311700
C	24.16235400	18.97516000	14.61545800
C	25.39691600	18.84145200	15.30005300
C	26.44188600	18.08122900	14.71550600
C	16.83072800	-0.85540300	14.81752800
C	15.85269300	-4.03615400	14.62524200
C	16.86697700	-4.75159000	15.31082200
C	18.15206900	-4.87810200	14.72419200
N	22.21115800	15.33343300	15.90029200
N	22.05658300	15.55173100	13.73489000
H	24.16088800	19.03721600	13.51030900
H	23.29844300	19.43616900	15.12073300
H	25.44420100	19.08715400	16.37842500
H	26.57121000	18.08457400	13.61680300
H	27.34851200	17.84999500	15.29839700
N	16.76093800	-0.02255300	15.89755200
N	16.53029600	-0.08170900	13.72814900
H	15.80804400	-4.08630800	13.52038600
H	14.90127900	-3.80661000	15.13150300
H	16.74178600	-4.96204300	16.39043200
H	18.24472500	-4.97097400	13.62573500
H	18.98538300	-5.30340400	15.30726900
C	22.68512300	15.56195300	17.26023200
C	21.10324600	14.57239900	15.50974700
C	22.16967300	16.23251900	12.44144500
C	21.02135300	14.69270800	14.12775500
C	16.39541900	1.27241300	15.50874400
C	16.97188500	-0.50576700	17.25744900
C	16.27775900	1.23850600	14.12484700
C	16.17861600	-0.67065300	12.43214300
H	21.86753600	15.96472300	17.89144700
H	23.06826800	14.62055900	17.70452800
H	23.50361500	16.30604700	17.20863100
C	20.14269500	13.81590600	16.28065900
H	21.16031600	16.53627500	12.09807400
H	22.63709600	15.59612800	11.66501900

H	22.79263800	17.13527500	12.59156800
C	20.05974400	13.95559800	13.34503100
C	16.09026800	2.45415100	16.28358900
H	16.08441700	-0.29075100	17.88631300
H	17.86913800	-0.03188500	17.70698200
H	17.12170500	-1.60176100	17.20297400
C	15.96703700	2.41138700	13.34549200
H	15.20081000	-0.26704700	12.09986800
H	16.93547600	-0.46455300	11.65077300
H	16.09429700	-1.76533500	12.57431000
C	20.26149500	13.53643500	17.65788300
C	19.01398400	13.29809000	15.53865600
C	20.18134600	13.73893800	11.95771200
C	18.99789700	13.32241800	14.09120600
C	15.53051700	3.56422400	15.54006100
C	16.32446100	2.58347200	17.66798700
C	15.48423100	3.54909800	14.09342600
C	16.17714700	2.51069400	11.95598800
C	19.30996100	12.77055800	18.36036900
H	21.13918000	13.88678000	18.20986700
C	17.98717000	12.61423600	16.28498200
C	19.28709700	12.91203800	11.24882500
H	21.03515100	14.17130800	11.41938600
C	18.01305400	12.58209700	13.34097400
C	15.14199600	4.73491200	16.28643400
C	15.94836000	3.73251300	18.39191300
H	16.83718400	1.78247700	18.21138700
C	15.08118800	4.70877500	13.33809400
C	15.87974500	3.68638000	11.23566900
H	16.62916200	1.66492000	11.42046900
C	19.56936300	12.42338900	19.84090400
C	18.16317500	12.35431400	17.66694500
C	16.89688700	12.08474900	15.50669100
C	19.55211100	12.60406600	9.76078300
C	18.19168800	12.38304300	11.94969200
C	16.93556200	12.01326800	14.11468500
C	14.67586000	5.85554100	15.50593400
C	15.31917700	4.77050500	17.69179900
C	16.29100500	3.83158700	19.89200300
C	14.61414900	5.83055600	14.11216600
C	15.29010700	4.74924400	11.93688600
C	16.22238100	3.76645400	9.73432300
C	18.53382600	11.42524500	20.39410800
C	19.50245800	13.72491300	20.67571400
C	20.97688100	11.79314400	19.99935800
H	17.42747800	11.74029900	18.19415800

N	15.74676000	11.39689700	15.90697600
C	20.94934100	11.95067300	9.61497000
C	19.51177600	13.92066500	8.94956100
C	18.51610100	11.62655600	9.17359200
H	17.48895600	11.74692400	11.40700300
N	15.82513000	11.24899100	13.73435700
N	14.33420800	7.15905400	15.88519400
H	14.99634000	5.64821000	18.25241800
C	15.75730800	5.12953500	20.52783900
C	15.69334000	2.62617400	20.65461300
C	17.83320400	3.83592600	20.03830400
N	14.18269200	7.10117700	13.71296300
H	15.05237900	5.66282800	11.38760800
C	15.82166600	5.11855300	9.11460300
C	15.48670100	2.63619900	8.97572900
C	17.75201000	3.60343700	9.55770400
H	17.50753000	11.85059400	20.39295900
H	18.52912400	10.47629400	19.81146800
H	18.78601500	11.17813500	21.44592600
H	19.69118800	13.50056800	21.74740700
H	20.26524900	14.46141000	20.34292400
H	18.50407200	14.20328800	20.59085500
H	21.08646800	10.86919700	19.39021200
H	21.78489900	12.49694800	19.70768500
H	21.14589900	11.52580400	21.06496900
C	15.19353200	11.33430700	17.25710400
C	15.09056400	10.88023200	14.82647700
H	21.75939500	12.60259500	10.00719500
H	20.99381400	10.97893500	10.15476200
H	21.16410200	11.75853900	8.54130000
H	19.69691200	13.71012200	7.87468900
H	18.52224500	14.41593200	9.04070700
H	20.28793200	14.63870000	9.29023700
H	18.52152900	10.65508900	9.71886900
H	17.48795200	12.04686600	9.19727500
H	18.76591300	11.41950200	8.11258600
C	15.39555700	10.92183300	12.37790100
C	14.02271200	7.91883800	14.79389800
C	14.38210700	7.74339600	17.22013300
H	14.65070100	5.20152900	20.45928800
H	16.20803400	6.02878200	20.04929500
H	16.02937100	5.15224100	21.60317200
H	16.11021000	1.65898700	20.30149500
H	15.92698300	2.71177700	21.73698000
H	14.58951200	2.58798400	20.54116500
H	18.30283500	2.94989500	19.55744800

H	18.11327600	3.83460200	21.11436100
H	18.26152400	4.74843200	19.56868500
C	13.82649800	7.53470400	12.36444300
H	14.72695400	5.29746300	9.17804400
H	16.35476900	5.96238300	9.60880400
H	16.10062800	5.12847400	8.04079800
H	15.71236000	2.70142000	7.89015400
H	15.80217200	1.62901900	9.32204200
H	14.38698400	2.71661300	9.10632900
H	18.13222100	2.67468200	10.03523100
H	18.00621100	3.56272700	8.47621200
H	18.28977600	4.46679100	10.00680800
H	15.33336000	12.30833100	17.76419400
H	15.67633000	10.52995800	17.85427500
H	14.10929000	11.12899500	17.17050100
Pd	13.29665300	9.85500600	14.80523200
H	16.07188700	10.16818900	11.91738500
H	15.36822400	11.83439800	11.75084100
H	14.37251300	10.50718400	12.44661500
H	13.70077000	7.21766400	17.91740300
H	15.42387100	7.71943300	17.61576400
H	14.04525900	8.79148400	17.12544900
H	13.24783200	6.74285600	11.85111300
H	14.73278200	7.78370800	11.76963300
H	13.18913900	8.43468200	12.46054400
C	11.99210100	11.59810000	14.99085000
C	11.30030900	10.55578600	14.32177800
C	11.19644400	9.28141100	14.93585000
H	11.92752000	11.66932800	16.09347400
H	12.20500500	12.54334600	14.46559300
H	11.09184800	10.65409400	13.23876300
H	11.09653200	9.21111600	16.03546300
H	10.79248700	8.42724300	14.36759100
C	22.49635200	4.94291100	12.32946300
C	23.68908700	5.64690200	12.78315500
C	21.68958600	5.86711700	11.54395900
C	21.86511600	4.00942200	13.17116700
C	23.61046000	7.01827200	12.28321400
C	24.20719700	5.39515400	14.07262900
C	22.37499900	7.15224500	11.51762500
C	20.28543100	5.81602000	11.62359500
C	22.39650900	3.75638200	14.49930300
C	20.41269200	3.93939000	13.24526400
C	24.04083800	8.08314600	13.09605700
C	24.66546600	6.50001000	14.90968500
C	23.53941800	4.44035000	14.94440500

C	21.63064700	8.34446400	11.57739600
C	19.51055300	7.05028000	11.67807800
C	19.63802400	4.83688300	12.48856100
C	21.27964200	3.52325500	15.40307900
C	20.04839700	3.63932600	14.62870500
C	24.56586000	7.81655000	14.42943400
C	23.27805500	9.32487900	13.15499000
C	24.31202900	6.21055000	16.29831800
C	23.61126600	4.93229300	16.31532200
C	22.09003900	9.44956500	12.41164700
C	20.17543500	8.29337100	11.65823500
C	18.37973600	6.83724100	12.57574100
C	18.46779500	5.47119200	13.08116100
C	21.34174300	4.00331500	16.72304800
C	18.92607800	4.26209400	15.20361200
C	24.12988500	8.89192500	15.31003800
C	23.33635300	9.82708100	14.52287300
C	23.88281200	7.25195400	17.15084500
C	22.52600600	4.72208100	17.18746200
C	20.92078100	10.08595100	13.00347300
C	19.73657200	9.36703700	12.53918100
C	17.95063800	7.87855900	13.42830800
C	18.13248500	5.19720400	14.41645900
C	20.17248800	4.63975700	17.31476800
C	18.98445500	4.76433800	16.57146600
C	23.79468200	8.61798400	16.64536800
C	22.21405200	10.44977600	15.09782500
C	22.75194200	7.03891400	18.04848100
C	22.08710000	5.79579200	18.06839000
C	20.98283400	10.56593300	14.32347400
C	18.65153200	9.15664900	13.41143600
C	17.59713100	7.58919200	14.81690400
C	17.69664500	6.27264800	15.29709900
C	20.63185800	5.74473000	18.14918600
C	18.22169400	6.00607500	16.63045700
C	22.62444700	9.25230300	17.23798900
C	21.84982300	10.14986400	16.48133300
C	21.97710200	8.27325000	18.10307700
C	19.86597000	10.33293400	15.22731400
C	18.72312800	9.64885000	14.78222400
C	18.05521800	8.69414700	15.65401900
C	19.88754300	6.93698900	18.20894100
C	18.65211500	7.07095300	17.44331900
C	20.39736600	10.07989900	16.55546300
C	20.57291800	8.22218700	18.18268500
C	18.57345700	8.44236200	16.94346500

C	19.76609100	9.14649100	17.39730600

Atom coordinates of [C_{70}@1’]$^{4+}$, [C202H156N16Pd4]

Pd	24.74921300	16.96728700	14.89815100
C	22.96821500	15.90738000	14.87656300
C	24.05660400	19.03468900	14.72293100
C	26.41465600	18.37029400	14.76286500
C	25.31032600	19.00667700	15.38485000
C	25.70016900	15.13915200	14.89957300
N	22.42147500	15.26593900	15.95159200
N	22.19775700	15.55579300	13.80174000
H	24.03039200	19.12788300	13.62031900
H	23.16069000	19.39507900	15.25370300
H	27.35074300	18.21549100	15.32425100
H	26.52213100	18.41330400	13.66275000
H	25.35243500	19.22742900	16.46881300
N	26.05002300	14.40114700	15.99721700
N	25.80235000	14.29727900	13.82998000
C	21.28782600	14.53319000	15.57552600
C	22.95126400	15.43628300	17.29942500
C	21.16236800	14.69863900	14.20029900
C	22.30875100	16.24064600	12.51213300
C	26.33493800	13.08221500	15.61983500
C	26.41303200	15.03540500	17.26995600
C	26.21571600	13.02040600	14.23687400
C	25.51898800	14.75448200	12.47401700
C	20.30606800	13.82306000	16.36295900
H	22.15888100	15.79991900	17.98349800
H	23.36647400	14.47859800	17.68285800
H	23.75661400	16.19405600	17.24480500
C	20.12183200	14.05494500	13.43631600
H	22.73384800	15.58573400	11.72563900
H	21.30733300	16.59116400	12.19113100
H	22.97291000	17.11467600	12.65367300
C	26.56543900	11.91284500	16.42758800
H	25.65988000	14.86699300	18.06401100
H	27.38970600	14.63589200	17.60934300
H	26.50453900	16.12366100	17.08599300
C	26.49461700	11.81352400	13.49056900
H	24.68236000	14.17504800	12.03111800
H	25.23667900	15.82322600	12.53808800
H	26.41812200	14.66264300	11.83247400
C	19.12846400	13.38151400	15.64992300
C	20.44923200	13.53237400	17.74024200
C	19.05678500	13.45300100	14.20654300
C	20.13043200	13.94859800	12.03033400
C	26.93825900	10.71412500	15.71635300
C	26.30913600	11.86859500	17.81191700

C	26.92615600	10.67202800	14.27048400
C	26.35189500	11.69495900	12.09168200
C	18.08060400	12.75547900	16.42300700
C	19.40908400	12.95773700	18.49243000
H	21.39867500	13.74874600	18.23700300
C	17.97534800	12.83947100	13.47436000
H	20.97384400	14.36162400	11.46334900
C	19.11443700	13.27618100	11.32450100
C	27.18347100	9.53088200	16.50336100
H	25.95939400	12.77457700	18.32275500
C	26.39186300	10.67157400	18.54839200
C	27.21858400	9.45081100	13.55809400
C	26.59812900	10.49089400	11.40575700
H	26.03643500	12.56038600	11.50187800
C	16.99746500	12.17508600	15.66645100
C	18.21486300	12.64102000	17.82386200
C	19.52790300	12.67951900	20.00481200
C	16.94206500	12.22306000	14.27298100
C	18.03774000	12.75759600	12.06182500
C	19.22021800	13.13651500	9.79301400
C	27.60069800	8.36416800	15.76510400
C	26.87718700	9.53016100	17.88801200
C	25.91763700	10.66123000	20.01524900
C	27.60769200	8.32202900	14.37066200
C	27.04326300	9.39103700	12.15440800
C	26.41047400	10.42478000	9.87648800
N	15.91553200	11.38307000	16.07474800
H	17.39373400	12.23326600	18.41818200
C	20.99346800	12.73713400	20.48103500
C	18.98343000	11.26975100	20.34515000
C	18.69720800	13.74168200	20.76243900
N	15.81838600	11.47427300	13.90013800
H	17.25296200	12.22739100	11.51910600
C	20.56997300	12.47221500	9.42988400
C	19.14674100	14.54213900	9.15095000
C	18.09311400	12.26300600	9.20898200
N	27.92075600	7.07121400	16.20027100
H	26.94488100	8.59303400	18.44822400
C	25.93976600	9.24588100	20.62442600
C	26.83323800	11.57929800	20.85876000
C	24.46185800	11.19324800	20.07923700
N	27.92306700	7.00178100	14.02261600
H	27.20382800	8.44446800	11.63482400
C	26.36856700	8.96798900	9.37144400
C	25.09112700	11.11438100	9.45070700
C	27.59899500	11.15912100	9.21093200

C	15.20237500	10.94265800	14.99707100
C	15.52685000	11.02968800	17.43619200
H	21.62715100	12.02629000	19.90378200
H	21.42451700	13.75735800	20.39091900
H	21.04627500	12.45554100	21.55317400
H	17.89575800	11.16727200	20.14372900
H	19.52065700	10.48273600	19.77481400
H	19.12823800	11.07024700	21.42799900
H	19.07263700	14.76600900	20.55501600
H	17.62761500	13.70178900	20.46319700
H	18.75472800	13.56339900	21.85774900
C	15.26921400	11.29701400	12.55922300
H	20.65564500	11.47249800	9.90646100
H	21.44165300	13.08310400	9.74672800
H	20.63905700	12.34085300	8.32897400
H	19.22814700	14.45997900	8.04605300
H	19.97335400	15.19590600	9.50331200
H	18.18551200	15.04293000	9.39216500
H	18.23355500	12.16491800	8.11290400
H	17.09067600	12.71233200	9.37485900
H	18.10501100	11.23863000	9.64671700
C	28.12069600	6.23597200	15.13764000
C	28.17201800	6.63972300	17.57338000
H	25.29334300	8.54247000	20.05290200
H	26.96993000	8.83177900	20.66395600
H	25.56023700	9.28697500	21.66617600
H	26.48855200	11.59338600	21.91482600
H	27.88379800	11.22056300	20.84149600
H	26.82121000	12.62537700	20.48488300
H	24.08012500	11.12502600	21.12108400
H	24.39151300	12.26032800	19.77430000
H	23.78526300	10.60484200	19.42051900
C	28.05893400	6.43184500	12.68582700
H	25.58471500	8.37864300	9.89997600
H	27.34533500	8.45456200	9.49869100
H	26.13546700	8.95693500	8.28691800
H	25.10018200	12.20706900	9.64772300
H	24.21317100	10.67191300	9.96895200
H	24.94824800	10.98938600	8.35672400
H	28.56537900	10.68389900	9.48198000
H	27.63910700	12.22363800	9.52816400
H	27.49465800	11.13348900	8.10490200
Pd	13.46369400	9.81776000	15.04715200
H	16.29864300	10.38901900	17.91890200
H	15.35724300	11.93924500	18.04541400
H	14.57380000	10.47356500	17.36914600

H	15.23194800	12.26765100	12.02895000
H	15.86875600	10.56557200	11.97148200
H	14.23367500	10.92509200	12.67375300
Pd	28.84333700	4.28937300	15.16318800
H	28.73797000	7.42016300	18.11719300
H	27.22130600	6.42106900	18.10868500
H	28.78982500	5.72283800	17.52984400
H	28.53675500	5.44149000	12.80008500
H	27.06310100	6.30949600	12.20036300
H	28.71137900	7.06575800	12.05525800
C	14.17148500	7.86611200	14.99248100
C	11.38504800	9.21048700	15.31778700
C	12.15083200	11.53798200	15.31269600
C	11.42978500	10.48400100	14.69444300
C	30.92445700	4.87394600	15.47665600
C	30.14321900	2.55378500	15.38357900
C	30.87708800	3.62330200	14.80873900
C	27.10013600	3.17476200	15.05525800
N	14.31607400	7.08170500	13.88241900
N	14.37970900	7.03768100	16.05875800
H	11.35035700	9.14630300	16.42229100
H	10.95778900	8.34896700	14.77894200
H	12.31634000	12.48388000	14.77121800
H	12.17063000	11.61228700	16.41680700
H	11.14715300	10.57427000	13.62791200
H	30.94978100	4.89752400	16.58295500
H	31.36427800	5.75031200	14.97303600
H	29.97572300	1.62849700	14.80801500
H	30.11183000	2.44179200	16.48407500
H	31.16996600	3.56812400	13.74247700
N	26.50953300	2.64521600	13.94400800
N	26.35608800	2.74237000	16.11481300
C	14.60873900	5.75909300	14.23925700
C	14.21830000	7.64344500	12.53944400
C	14.67715100	5.73782400	15.63150300
C	14.20546800	7.49906400	17.43252400
C	25.37058600	1.90402100	14.28937800
C	27.08911800	2.81911500	12.61559100
C	25.28328300	1.95161800	15.68101900
C	26.68771600	3.13302500	17.48095800
C	14.94578300	4.61686900	13.42207600
H	13.97369300	8.71423900	12.65483400
H	15.18664600	7.54141500	11.99879600
H	13.40953100	7.15605400	11.95996600
C	15.14609800	4.58810200	16.36795500
H	13.66538600	6.73526300	18.02354600

H	15.18623000	7.72415800	17.91063000
H	13.58929200	8.41735000	17.40250400
C	24.35794000	1.28130800	13.46820200
H	27.11572600	1.85062700	12.08034400
H	26.51395700	3.56388600	12.02024100
H	28.12836400	3.17422800	12.75098900
C	24.19306900	1.35718500	16.41527900
H	25.88637600	3.77223500	17.91529900
H	26.84720500	2.24286100	18.12091200
H	27.63171700	3.70709200	17.43587800
C	15.34908600	3.42685200	14.13632500
C	14.95417300	4.62418200	12.01074700
C	15.39926600	3.40436700	15.58183700
C	15.48704300	4.60512100	17.74393900
C	23.28243300	0.63388500	14.18122000
C	24.29813600	1.40277500	12.05639200
C	23.18902900	0.68957200	15.62490600
C	24.01941600	1.50079500	17.81292200
C	15.79777000	2.29780600	13.35178200
C	15.43689600	3.54290100	11.25239300
H	14.60403000	5.50437000	11.46448600
C	15.82229800	2.22125300	16.28929100
H	15.41393100	5.54203700	18.30387700
C	15.99017400	3.47059700	18.40598300
C	22.23314400	0.02983200	13.39015400
H	25.06899700	1.97543800	11.53444500
C	23.23272000	0.88381500	11.30242200
C	22.02102800	0.19140800	16.32118400
C	22.85519600	1.08047100	18.47091200
H	24.79346600	1.99124000	18.40353900
C	16.14787300	1.10994800	14.09615700
C	15.86739600	2.39797800	11.94117600
C	15.50002500	3.67472600	9.71749900
C	16.06162600	1.05362300	15.48242000
C	16.09129700	2.27476300	17.67147000
C	16.42884000	3.47867100	19.88439500
C	21.19465900	-0.64592800	14.13076900
C	22.23201900	0.16750700	11.98773500
C	23.12509900	1.05380900	9.77312300
C	21.06457000	-0.52677300	15.50998200
C	21.86672900	0.43536500	17.70257000
C	22.65302500	1.25899000	19.99010300
N	16.55160300	-0.16990600	13.68529200
H	16.25324300	1.55708900	11.36070600
C	16.42028400	4.86675100	9.35765800
C	16.07430300	2.41288500	9.04624800

C	14.07974900	3.92507400	9.15827800
N	16.35117300	-0.26463500	15.85852300
H	16.45695900	1.37506700	18.18153000
C	16.44787600	4.89835900	20.48366700
C	15.44443100	2.60923300	20.70298500
C	17.85343700	2.87866600	20.00368300
N	20.15641100	-1.48441300	13.70087000
H	21.40182200	-0.25005100	11.40613700
C	21.72731700	1.60150200	9.38721800
C	23.32047000	-0.33223700	9.11163300
C	24.18920500	2.02117300	9.21898400
N	19.93158700	-1.27237000	15.85933000
H	20.95555700	0.10981500	18.21380200
C	21.28982700	1.92690200	20.28795000
C	23.75123400	2.13645700	20.62246400
C	22.68611800	-0.14132300	20.64973200
C	16.67172100	-1.00872100	14.75521400
C	16.79505600	-0.63509800	12.32388100
H	17.44944900	4.69468500	9.74220000
H	16.05154500	5.82769400	9.78070200
H	16.47554800	4.98268500	8.25393800
H	15.44750500	1.51731800	9.24585300
H	17.11373400	2.20782600	9.38543800
H	16.10321500	2.56055800	7.94714200
H	13.63527900	4.86324500	9.55252600
H	13.39636000	3.08762500	9.41199000
H	14.12190200	4.01493400	8.05199200
C	15.99233400	-0.89377000	17.13508500
H	17.13378300	5.57340500	19.92543500
H	15.43415700	5.35259900	20.49859200
H	16.79921700	4.84808500	21.53486500
H	15.75642400	2.58171200	21.76906200
H	14.41329700	3.01805400	20.65308600
H	15.41779000	1.56345400	20.32835300
H	18.22143300	2.98061100	21.04779200
H	17.86858500	1.79433400	19.76042500
H	18.57402000	3.38981200	19.32806800
C	19.38069800	-1.86728500	14.76049100
C	20.03228700	-2.11742500	12.38649100
H	21.51697600	2.57846400	9.87403800
H	20.91083900	0.89835200	9.65504000
H	21.68174100	1.74778100	8.28709800
H	23.24497600	-0.24117300	8.00689500
H	22.54551200	-1.05458300	9.44765500
H	24.31626300	-0.75666000	9.35906700
H	24.04383200	2.14201300	8.12579900

H	25.21933000	1.63452400	9.37230300
H	24.11130100	3.02820800	9.68756900
C	19.40906300	-1.50604300	17.20042400
H	21.24968600	2.96223900	19.88622300
H	20.43533300	1.35722200	19.86631000
H	21.13868500	1.98049500	21.38772800
H	24.75425100	1.66358400	20.55619000
H	23.79354100	3.14031000	20.13969200
H	23.52949200	2.28581600	21.69927600
H	21.86484600	-0.78660000	20.26897300
H	23.64886300	-0.65638700	20.44754300
H	22.56424200	-0.04819800	21.75015100
Pd	17.56765400	-2.86603400	14.73303800
H	17.61570400	-0.05679500	11.85159300
H	17.08211100	-1.70283100	12.38584900
H	15.87699500	-0.54862400	11.70867900
H	15.90482100	-1.98335500	16.95674900
H	16.74387400	-0.71793700	17.92832200
H	15.01391500	-0.49651500	17.47271600
H	19.61505500	-1.42618300	11.62729700
H	21.02694400	-2.47163600	12.04919200
H	19.35524100	-2.98611300	12.49710000
H	20.20020300	-1.92021600	17.85743500
H	19.01568300	-0.56476700	17.63878900
H	18.58902600	-2.24484600	17.11306100
C	18.18860800	-4.95145500	14.51594400
C	15.85483800	-4.20417300	14.53901300
C	16.92641800	-4.89545700	15.15978200
H	18.22983000	-5.01685500	13.41178100
H	19.06291200	-5.35629000	15.05065400
H	14.91666700	-4.03021500	15.09106900
H	15.76256400	-4.21615000	13.43664800
H	16.85962900	-5.14202700	16.23693300
C	21.22214500	9.91286000	17.44661600
C	22.25834400	10.27353600	16.48883300
C	21.42331800	8.85232700	18.35148000
C	19.94943100	10.03811800	16.75981300
C	23.46377900	9.55714500	16.46875200
C	21.62160200	10.63913500	15.23225600
C	22.67829700	8.11355200	18.33303100
C	20.34742200	7.90615400	18.61299400
C	20.19783200	10.48274600	15.40356600
C	18.90337500	9.14043400	17.01860700
C	24.05585700	9.19104500	15.19426100
C	23.67695000	8.46364800	17.40616400
C	22.23843900	10.38059600	13.96731700

C	22.37948800	6.71088700	18.58497200
C	20.93837900	6.58349400	18.75388100
C	19.10639500	8.05305400	17.96110800
C	19.35249500	10.08905700	14.32189400
C	18.08026900	8.65719800	15.92114200
C	24.62621000	7.87519700	15.33508700
C	23.50574800	9.62935900	13.94768700
C	24.40302400	7.42570600	16.69618300
C	21.36007900	10.26601500	12.84119900
C	23.09739000	5.70637700	17.90745300
C	20.26728600	5.45864700	18.23731100
C	18.42196700	6.88869100	17.42151700
C	19.93530900	10.12334900	13.01845100
C	18.25564500	9.14661300	14.58752400
C	17.79121600	7.26288500	16.16815100
C	24.68044700	6.96179700	14.23850700
C	23.80275800	8.81864100	12.80819700
C	24.12538700	6.07288500	16.94230800
C	21.73279600	9.50594500	11.66182800
C	22.39315900	4.55783600	17.36159900
C	21.00601900	4.43053200	17.52476000
C	18.99552400	5.61555500	17.55363800
C	19.42064300	9.30351400	11.93666900
C	17.82591000	8.28656000	13.52865600
C	17.67030000	6.33238900	15.08900100
C	24.39674400	5.54109300	14.49226200
C	24.38844400	7.50591800	12.94943700
C	22.94031400	8.79473000	11.64365100
C	24.06881800	5.14210300	15.82839400
C	20.53504300	8.91623600	11.08694300
C	22.99013300	4.22102900	16.08497500
C	20.18442500	3.93670000	16.43329600
C	18.94148800	4.67286200	16.45022700
C	18.38160000	8.39244900	12.18810700
C	17.55294300	6.89165100	13.78036900
C	18.25725200	4.99030200	15.23501800
C	23.81207100	4.77037400	13.43976900
C	23.89414100	6.67252600	11.86883300
C	22.99672100	7.46945900	11.04873700
C	20.58265300	7.62897700	10.52126500
C	22.21737000	3.66167900	15.02206900
C	20.76593500	3.50065100	15.20006700
C	18.42586900	7.06211200	11.59975700
C	17.91526400	6.13629700	12.59628600
C	18.68873100	4.30110500	14.05835300
C	22.73126800	3.85216700	13.70637400

C	23.60977800	5.31846100	12.11051400
C	21.83795800	6.89160500	10.50117000
C	19.50856100	6.68240400	10.78243000
C	19.92949500	3.56116000	14.04000300
C	18.47926200	4.85857600	12.73390700
C	21.87430800	3.80916000	12.54029200
C	22.41327800	4.71798700	11.54265100
C	21.54012700	5.48905200	10.75270800
C	20.09998400	5.35856500	10.92694500
C	20.48921300	3.66935200	12.70307900
C	19.58484700	4.45784400	11.88116400

Atom coordinates of [(NTCDI)2(corannulene)@1’]$^{4+}$, [C180H178N20O8Pd4]

C	12.59040000	9.30060000	17.20330000
C	13.29290000	10.34210000	17.83720000
C	11.20650000	8.98120000	17.52560000
H	13.08250000	8.78100000	16.36340000
C	12.67800000	11.17440000	18.86360000
H	14.30870000	10.58720000	17.46900000
C	10.72130000	9.67880000	18.63380000
C	10.17520000	8.25290000	16.80410000
C	12.99970000	12.50520000	19.34810000
C	11.42850000	10.72070000	19.28350000
C	9.34480000	9.83340000	18.93510000
C	8.80790000	8.42640000	17.09380000
H	10.44940000	7.62840000	15.93200000
C	12.07160000	13.30530000	20.03340000
H	13.95560000	12.95810000	19.04890000
C	10.48790000	11.52180000	19.98650000
C	9.19750000	10.96860000	19.77430000
C	8.32620000	9.31600000	18.13770000
H	8.07570000	7.95270000	16.42660000
H	12.35560000	14.35030000	20.25510000
C	10.71010000	12.86080000	20.30410000
C	8.01650000	11.70680000	19.85410000
C	7.03260000	9.94090000	18.36900000
C	9.48830000	13.60340000	20.58720000
C	8.21070000	13.05430000	20.38070000
C	6.88560000	11.07540000	19.18260000
H	6.15160000	9.58750000	17.80370000
H	9.55450000	14.66020000	20.90120000
H	7.33470000	13.70430000	20.55380000
H	5.89130000	11.55330000	19.22620000
H	15.15360000	9.57820000	15.19560000
C	15.80150000	8.70410000	15.44570000
C	17.24760000	9.15230000	15.76440000
H	15.35310000	8.18430000	16.32640000
H	15.80020000	8.00700000	14.57940000
C	17.16200000	10.19580000	16.89450000
C	17.89230000	9.74830000	14.49080000
C	18.07090000	7.91350000	16.18130000
C	16.91170000	11.55010000	16.59750000
C	17.26090000	9.82170000	18.24880000
H	18.04840000	8.93840000	13.74780000
H	18.88380000	10.19590000	14.71670000
H	17.25740000	10.51620000	14.00400000
H	18.18770000	7.24100000	15.30610000

H	17.58330000	7.32080000	16.98300000
H	19.08810000	8.20280000	16.52260000
C	16.74100000	12.51750000	17.61650000
H	16.79240000	11.85020000	15.54710000
C	17.08080000	10.75780000	19.29450000
H	17.43780000	8.76670000	18.49940000
C	16.55570000	13.92650000	17.38420000
C	16.73360000	12.12470000	19.00150000
C	17.22090000	10.45570000	20.69530000
N	16.78530000	14.68710000	16.23690000
C	16.11170000	14.80660000	18.36840000
C	16.31300000	13.03970000	20.04140000
C	16.74700000	11.29820000	21.69550000
N	17.82550000	9.37190000	21.33550000
C	16.47130000	15.99890000	16.45680000
C	17.49290000	14.24280000	15.03310000
N	16.04340000	16.06710000	17.75520000
C	15.87820000	14.38150000	19.73120000
C	16.15930000	12.58640000	21.40490000
N	17.05170000	10.67030000	22.91070000
C	17.72580000	9.50030000	22.69150000
C	18.66610000	8.34580000	20.71200000
Pd	16.50420000	17.43340000	14.97050000
H	18.32100000	13.56780000	15.32600000
H	16.82010000	13.71300000	14.32550000
H	17.91760000	15.14100000	14.54710000
C	15.71930000	17.33860000	18.40020000
C	15.20230000	15.14890000	20.71070000
C	15.45870000	13.38210000	22.33440000
C	16.86960000	11.22650000	24.25130000
Pd	18.34270000	8.07150000	24.03520000
H	18.06600000	7.51100000	20.28910000
H	19.27170000	8.80770000	19.90780000
H	19.34680000	7.95430000	21.49190000
C	14.94240000	16.48820000	14.00070000
C	17.96800000	18.88070000	14.28280000
H	14.63810000	17.40420000	18.64120000
H	16.31320000	17.45630000	19.32840000
H	15.98500000	18.14760000	17.69400000
C	14.94380000	14.65150000	22.00130000
H	14.81430000	16.13640000	20.43970000
H	15.26700000	12.99290000	23.34210000
H	17.23840000	12.27140000	24.27890000
H	15.80290000	11.20190000	24.55660000
H	17.46590000	10.61070000	24.95210000
C	16.82950000	6.81830000	23.38870000

C	19.58550000	8.09500000	25.81340000
N	13.63460000	16.49080000	14.39640000
N	14.99760000	15.68790000	12.89310000
C	16.73020000	19.14540000	13.64310000
C	18.02180000	18.83210000	15.69910000
H	18.80210000	18.44980000	13.69570000
C	14.14620000	15.44100000	23.05950000
N	15.50560000	6.96770000	23.68820000
N	16.91270000	5.71500000	22.58620000
C	20.11950000	9.01670000	24.87630000
C	19.38270000	6.74550000	25.42820000
H	19.08050000	8.47660000	26.72170000
C	12.85810000	15.70650000	13.53720000
C	13.18090000	17.22810000	15.57560000
C	13.72930000	15.17260000	12.58980000
C	16.22560000	15.52160000	12.11570000
H	16.64130000	19.04090000	12.54890000
H	16.00770000	19.83720000	14.11520000
H	17.37980000	19.51820000	16.28410000
H	18.93860000	18.48750000	16.20430000
C	12.97190000	14.58140000	23.58900000
C	15.09280000	15.79010000	24.23410000
C	13.56220000	16.74910000	22.49340000
C	14.74830000	5.94520000	23.10520000
C	15.03060000	8.06190000	24.53410000
C	15.64540000	5.14670000	22.39670000
C	18.20180000	5.18470000	22.14080000
H	20.87410000	8.67540000	24.14310000
H	20.14760000	10.09240000	25.11700000
H	18.83570000	6.05940000	26.09490000
H	20.09980000	6.26840000	24.73310000
C	11.45500000	15.37980000	13.58210000
H	12.60510000	18.12990000	15.28300000
H	12.55090000	16.58330000	16.22470000
H	14.08210000	17.55400000	16.12820000
C	13.30230000	14.20500000	11.60460000
H	16.52670000	14.45370000	12.08310000
H	16.08290000	15.90450000	11.08530000
H	17.01450000	16.12050000	12.60890000
H	12.26630000	14.30820000	22.77500000
H	12.41070000	15.15840000	24.35430000
H	13.31480000	13.64140000	24.06870000
H	15.49780000	14.87570000	24.71870000
H	14.53990000	16.36490000	25.00720000
H	15.94970000	16.40670000	23.88910000
H	14.35620000	17.45250000	22.16310000

H	12.97540000	17.26160000	23.28290000
H	12.87720000	16.55560000	21.63720000
C	13.33380000	5.66670000	23.16390000
H	15.91500000	8.66480000	24.81100000
H	14.29440000	8.69590000	23.99200000
H	14.57340000	7.66960000	25.46430000
C	15.22490000	3.99660000	21.63080000
H	18.33720000	4.14710000	22.50610000
H	18.27860000	5.21010000	21.03390000
H	18.99040000	5.82260000	22.58190000
C	11.03500000	14.31070000	12.70600000
C	10.51200000	16.04380000	14.39830000
C	11.94020000	13.73910000	11.73030000
C	14.14130000	13.67810000	10.59400000
C	12.88200000	4.53840000	22.38630000
C	12.40960000	6.41090000	23.93800000
C	13.80680000	3.71770000	21.63330000
C	16.10130000	3.19910000	20.85860000
C	9.66930000	13.86090000	12.82610000
C	9.16950000	15.62940000	14.48150000
H	10.80830000	16.94530000	14.94500000
C	11.50880000	12.68890000	10.83320000
C	13.72770000	12.63970000	9.74580000
H	15.15250000	14.07140000	10.47480000
C	11.46570000	4.25520000	22.39960000
C	11.05670000	6.04540000	24.05150000
H	12.75480000	7.27550000	24.50870000
C	13.34840000	2.58900000	20.85370000
C	15.64640000	2.12420000	20.07800000
H	17.16240000	3.45120000	20.81930000
C	9.28050000	12.78580000	11.94420000
C	8.79020000	14.49880000	13.73590000
C	8.13510000	16.49580000	15.23290000
C	10.13970000	12.25170000	10.98470000
C	12.41800000	12.14690000	9.89830000
C	14.66650000	11.99230000	8.70640000
C	11.03030000	3.20300000	21.51540000
C	10.60480000	4.97160000	23.26200000
C	10.13290000	6.67390000	25.11730000
C	11.91690000	2.39270000	20.80970000
C	14.27610000	1.81410000	20.12350000
C	16.60590000	1.30520000	19.19130000
N	8.05980000	12.11620000	11.79590000
H	7.76250000	14.14230000	13.83380000
C	7.92240000	17.76510000	14.36690000
C	6.78110000	15.77720000	15.39950000

C	8.61250000	16.93080000	16.63720000
N	9.39670000	11.29600000	10.27860000
H	12.12050000	11.29400000	9.28100000
C	15.96560000	12.79790000	8.50910000
C	15.04420000	10.58000000	9.21680000
C	13.95740000	11.87590000	7.33610000
N	9.74250000	2.80750000	21.13760000
H	9.56320000	4.65450000	23.37270000
C	8.70200000	6.94300000	24.59960000
C	10.69370000	8.00170000	25.66750000
C	10.05280000	5.64760000	26.27750000
N	11.12710000	1.50840000	20.06160000
H	13.92480000	0.96890000	19.52770000
C	15.90540000	0.87590000	17.87820000
C	17.86190000	2.12760000	18.82340000
C	17.03490000	0.03860000	19.96890000
C	8.12030000	11.22410000	10.76310000
C	6.83510000	12.30720000	12.57280000
H	8.85600000	18.36270000	14.29030000
H	7.13970000	18.40720000	14.82470000
H	7.59950000	17.49710000	13.33850000
H	6.27570000	15.60780000	14.42450000
H	6.10420000	16.41110000	16.00880000
H	6.89200000	14.79650000	15.91470000
H	8.61200000	16.08770000	17.35660000
H	7.91710000	17.70400000	17.02690000
H	9.63220000	17.36920000	16.64330000
C	9.80910000	10.56540000	9.07960000
H	16.58010000	12.82670000	9.43490000
H	15.75660000	13.83960000	8.18440000
H	16.58140000	12.31620000	7.72230000
H	15.65560000	10.04470000	8.45900000
H	14.14050000	9.96250000	9.42790000
H	15.65690000	10.66450000	10.14420000
H	13.62000000	12.87010000	6.97480000
H	13.07800000	11.19880000	7.36770000
H	14.66220000	11.45800000	6.58710000
C	9.79810000	1.76140000	20.26080000
C	8.47320000	3.39360000	21.56730000
H	8.67180000	7.76060000	23.85300000
H	8.06460000	7.24700000	25.45640000
H	8.22340000	6.05300000	24.14040000
H	11.62830000	7.85090000	26.24920000
H	9.95310000	8.45120000	26.36060000
H	10.89860000	8.73780000	24.85780000
H	9.58410000	4.69770000	25.94130000

H	9.43900000	6.06110000	27.10630000
H	11.06380000	5.41190000	26.67290000
C	11.58840000	0.37090000	19.26530000
H	15.41100000	1.73130000	17.36450000
H	15.13250000	0.09720000	18.04820000
H	16.65160000	0.43510000	17.18540000
H	18.45940000	1.57840000	18.06740000
H	18.52560000	2.28980000	19.69890000
H	17.59890000	3.12700000	18.40490000
H	16.15190000	-0.58220000	20.23320000
H	17.56120000	0.30720000	20.90930000
H	17.72030000	-0.57920000	19.34970000
Pd	6.54180000	10.06270000	10.09270000
H	7.02330000	12.14970000	13.65630000
H	6.41880000	13.32190000	12.41010000
H	6.09930000	11.56600000	12.20890000
H	10.16780000	11.27310000	8.30540000
H	10.60510000	9.82970000	9.31760000
H	8.91870000	10.03560000	8.69270000
Pd	8.18420000	0.80800000	19.39170000
H	8.24440000	3.12280000	22.61820000
H	8.49780000	4.50000000	21.46810000
H	7.68080000	2.97530000	20.91910000
H	12.16980000	0.71470000	18.38420000
H	12.21060000	-0.30270000	19.88780000
H	10.69200000	-0.18360000	18.92800000
C	6.75580000	8.79850000	11.70110000
C	4.95090000	10.43880000	8.66650000
C	7.88510000	2.41890000	18.13830000
C	7.58940000	-1.27890000	19.36800000
N	5.96840000	8.64870000	12.80920000
N	7.85020000	8.00950000	11.90730000
C	6.13390000	11.05860000	8.18990000
C	4.96390000	9.05410000	8.97440000
H	4.12880000	11.07240000	9.05180000
N	6.91300000	3.38240000	18.14760000
N	8.77770000	2.79960000	17.17670000
C	6.50070000	-0.50090000	18.89800000
C	8.16090000	-0.98300000	20.63190000
H	8.15130000	-1.90930000	18.65180000
C	6.55310000	7.74840000	13.71290000
C	4.63820000	9.25020000	12.90030000
C	7.76430000	7.36380000	13.14160000
C	8.82860000	7.75000000	10.84820000
H	6.17760000	12.15500000	8.08750000
H	6.81310000	10.48810000	7.52780000

H	5.58120000	8.36480000	8.36830000
H	4.09730000	8.59330000	9.47710000
C	7.17630000	4.35990000	17.17770000
C	5.71320000	3.26530000	18.97490000
C	8.37800000	3.99540000	16.58070000
C	9.86490000	1.92740000	16.72610000
H	5.75320000	-0.13040000	19.62530000
H	6.14150000	-0.62410000	17.86350000
H	9.09500000	-1.47870000	20.94460000
H	7.51340000	-0.62600000	21.45450000
C	6.08360000	7.20170000	14.96640000
H	3.88520000	8.47150000	13.13360000
H	4.60570000	10.04290000	13.67620000
H	4.40700000	9.69600000	11.91360000
C	8.68250000	6.46330000	13.78730000
H	9.76370000	8.33190000	10.99560000
H	9.06350000	6.66820000	10.82250000
H	8.36090000	8.03540000	9.88680000
C	6.41030000	5.50090000	16.72770000
H	5.68700000	4.04900000	19.75990000
H	4.80560000	3.34280000	18.34330000
H	5.73680000	2.26930000	19.45560000
C	8.99720000	4.77180000	15.53730000
H	10.84660000	2.24990000	17.13260000
H	9.63350000	0.90190000	17.07030000
H	9.90330000	1.93510000	15.61910000
C	6.86120000	6.10420000	15.49290000
C	4.98730000	7.71290000	15.70310000
C	8.14510000	5.75370000	14.91880000
C	10.03610000	6.29000000	13.40460000
C	5.30790000	6.04720000	17.41690000
C	10.34750000	4.64150000	15.14130000
C	4.59380000	7.16390000	16.93710000
H	4.45560000	8.59190000	15.32520000
C	10.88950000	5.40370000	14.08680000
H	10.45180000	6.91370000	12.60180000
H	5.00180000	5.61390000	18.37610000
H	11.00970000	3.97360000	15.71010000
C	3.38980000	7.69810000	17.74060000
C	12.39650000	5.33940000	13.77440000
C	2.24790000	6.65730000	17.62990000
C	2.88260000	9.04900000	17.19910000
C	3.75460000	7.88590000	19.23320000
C	12.70410000	5.72610000	12.31090000
C	13.09290000	6.35000000	14.71970000
C	12.96990000	3.92420000	14.01760000

H	1.95060000	6.49810000	16.57170000
H	1.35760000	7.00940000	18.19350000
H	2.55450000	5.67640000	18.05360000
H	3.68240000	9.82360000	17.20930000
H	2.05210000	9.41250000	17.83810000
H	2.48470000	8.96250000	16.16560000
H	4.02330000	6.93070000	19.72980000
H	2.87400000	8.29210000	19.77400000
H	4.59970000	8.59400000	19.36930000
H	12.47710000	6.78800000	12.08540000
H	12.14060000	5.08320000	11.60110000
H	13.78620000	5.57690000	12.11360000
H	14.19690000	6.27350000	14.61600000
H	12.82330000	6.16480000	15.78820000
H	12.78120000	7.39260000	14.46470000
H	12.40340000	3.16130000	13.44200000
H	12.97160000	3.63210000	15.08640000
H	14.02400000	3.89440000	13.67060000
H	14.30230000	10.25370000	11.72920000
N	13.71980000	10.53630000	12.52750000
C	14.25270000	11.54670000	13.32950000
C	12.41870000	10.03080000	12.51610000
C	13.34670000	12.03850000	14.40250000
O	15.37830000	11.98630000	13.12910000
C	11.54050000	10.51150000	13.61830000
O	12.04490000	9.28020000	11.62260000
C	12.01670000	11.53120000	14.49550000
C	13.77950000	13.05310000	15.26200000
C	10.22190000	10.05320000	13.71040000
C	11.10910000	12.12000000	15.43510000
C	12.89570000	13.61820000	16.20640000
H	14.80300000	13.44230000	15.14550000
C	9.32530000	10.62920000	14.63930000
H	9.88360000	9.27510000	13.00920000
C	9.75320000	11.67030000	15.47070000
C	11.56390000	13.19050000	16.26240000
H	13.23110000	14.42440000	16.88490000
H	8.27580000	10.28570000	14.70600000
C	8.76830000	12.38660000	16.33210000
C	10.59760000	13.90180000	17.14820000
N	9.30600000	13.37250000	17.16010000
O	7.56530000	12.18140000	16.28820000
O	10.88850000	14.89540000	17.79450000
H	8.62850000	13.85940000	17.76190000
H	15.39230000	4.01070000	17.78750000
N	14.74220000	4.70610000	18.17920000

C	15.34460000	5.75470000	18.88170000
C	13.38920000	4.36510000	18.11570000
C	14.40130000	6.68420000	19.55740000
O	16.56430000	5.84990000	18.94910000
C	12.46550000	5.31380000	18.79480000
O	13.02230000	3.33890000	17.55550000
C	13.00250000	6.41030000	19.53420000
C	14.89520000	7.77570000	20.27820000
C	11.08630000	5.08760000	18.76580000
C	12.12220000	7.21640000	20.32280000
C	14.02720000	8.59260000	21.03600000
H	15.97880000	7.95400000	20.27900000
C	10.20740000	5.91420000	19.50450000
H	10.70390000	4.22590000	18.19530000
C	10.72260000	6.92930000	20.31640000
C	12.66230000	8.29100000	21.09230000
H	14.40830000	9.45960000	21.60710000
H	9.11370000	5.75560000	19.47410000
C	9.80030000	7.71420000	21.19180000
C	11.76180000	9.08520000	21.98410000
N	10.41240000	8.73180000	21.93120000
O	8.60820000	7.48200000	21.28420000
O	12.16260000	9.96050000	22.73340000
H	9.78590000	9.29430000	22.52140000
